# Oral Health and Nutraceutical Agents

**DOI:** 10.3390/ijms25179733

**Published:** 2024-09-09

**Authors:** Mariantonietta Leo, Floriana D’Angeli, Carlo Genovese, Antonella Spila, Chiara Miele, Dania Ramadan, Patrizia Ferroni, Fiorella Guadagni

**Affiliations:** 1 Department of Promotion of Human Sciences and Quality of Life, San Raffaele Roma Open University, 00166 Rome, Italy; mariantonietta.leo@uniroma5.it (M.L.); floriana.dangeli@unikore.it (F.D.); antonella.spila@sanraffaele.it (A.S.); chiara.miele@sanraffaele.it (C.M.); dania.ramadan@uniroma5.it (D.R.); fiorella.guadagni@uniroma5.it (F.G.); 2 Department of Medicine and Surgery, “Kore” University of Enna, Contrada Santa Panasia, 94100 Enna, Italy; carlo.genovese@unikore.it; 3 Nacture S.r.l., Spin-Off University of Catania, Via Santa Sofia 97, 95123 Catania, Italy; 4 InterInstitutional Multidisciplinary Biobank (BioBIM), IRCCS San Raffaele, 00166 Rome, Italy

**Keywords:** oral health, nutraceuticals, antioxidant activity, antimicrobial activity, anticancer activity, oral diseases

## Abstract

Oral health is essential for both overall health and quality of life. The mouth is a window into the body’s health, and nutrition can strongly impact the state of general and oral health. A healthy diet involves the synergistic effect of various nutraceutical agents, potentially capable of conferring protective actions against some inflammatory and chronic-degenerative disorders. Nutraceuticals, mostly present in plant-derived products, present multiple potential clinical, preventive, and therapeutic benefits. Accordingly, preclinical and epidemiological studies suggested a protective role for these compounds, but their real preventive and therapeutic effects in humans still await confirmation. Available evidence suggests that plant extracts are more effective than individual constituents because they contain different phytochemicals with multiple pharmacological targets and additive/synergistic effects, maximizing the benefits for oral health. Moreover, nutritional recommendations for oral health should be personalized and aligned with valid suggestions for overall health. This review is aimed to: introduce the basic concepts of nutraceuticals, including their main food sources; examine the logic that supports their relationship with oral health, and summarize and critically discuss clinical trials testing the utility of nutraceuticals in the prevention and treatment of oral diseases.

## 1. Introduction

Oral disorders represent a major public health concern worldwide, due to their ability to negatively impact the quality of life and deteriorate daily activities [1]. Although their etiology often recognizes a microbial component, multiple factors have been called into question, including a wide range of potentially modifiable and non-modifiable risk factors (e.g., smoking, alcohol consumption, stress, poor oral hygiene, systemic health, genetics, and epigenetic factors), all significantly contributing to the severity and progression of oral diseases [2,3]. Dietary habits, in particular, are a widely modifiable factor in oral health, which can be positively or negatively impacted by diet depending on the physical and chemical characteristics of the foods we consume.

This concept became strengthened over time [4]. Indeed, it is currently widely acknowledged that a balanced diet, rich in nutrients such as vitamins, minerals, proteins, and healthy fats, supports vital functions of the organism, boosts immunity, and reduces the risk of chronic inflammatory diseases at both the systemic and oral levels [5,6]. On the other hand, poor dietary habits, including excessive consumption of processed foods, sugars, and unhealthy fats, can lead to various pathological conditions, including malnutrition, increased inflammation, and metabolic disorders [5,7,8]. Thus, maintaining a healthy diet is essential for promoting health and preventing illness [9]. In this regard, nutraceuticals, bioactive compounds found in foods, could play a significant role [10].

Nutraceutical substances are produced by plants, mostly for defensive purposes. They are secondary metabolites, or phytochemical compounds, present in plant tissues, which have different pharmacological, molecular, and biochemical targets on both healthy and altered cells and tissues [11,12]. The rationale behind the use of nutraceutical substances is accompanied by the ever-growing use of herbs with medicinal properties in the treatment of different pathological processes, with efficacy not always established [13]. In this context, the exploration of nutraceuticals has gained considerable interest in the management of oral diseases. These natural products offer, in fact, a promising complementary approach to conventional dental therapies, particularly in the prevention and treatment of conditions such as periodontitis, dental caries, and oral cancers [14,15].

To obtain useful recommendations for clinicians, the most relevant information from the scientific literature supporting the use of nutraceutical substances in the prevention and treatment of common oral pathologies was extracted by searching the most acknowledged databases (PubMed, Scopus, and Web of Science). The inclusion criteria regarded relevant research studies, among those published in English as to July 2024. For the selection of the search terms, we referred to previous literature reviews and the keywords of leading papers on the topic of interest (keywords: oral health; nutraceuticals; antioxidant activity; antimicrobial activity; anticancer activity; oral diseases).

## 2. Classes of Nutraceuticals and Effects on Oral Health: General Overview

One of the first studies correlating an increased risk of oral cancer with reduced intake of fruits and vegetables, regardless of demographic characteristics, body weight, tobacco, and alcohol use, dates back thirty years [16]. In 2011, the INHANCE consortium (International Head and Neck Cancer Epidemiology) conducted a comprehensive weighted analysis of 22 case-control studies, involving 14,520 cases and 22,737 controls. The conclusions were that a high intake of fruits and vegetables was associated with a reduced risk of head and neck tumors, while, conversely, the consumption of red meat increased the risk [17]. Regarding oral health, an Italian study reexamined epidemiological data on the effects of certain foods and nutrients on the development of oropharyngeal cancer: 6 cohort studies and 40 case-control studies suggested an inverse association between oral carcinoma and the consumption of fruits and vegetables, with a relative risk ranging from 0.52 to 0.78 [18].

## 3. Classes of Nutraceuticals and Food Sources

Among the main groups of nutraceutical substances are phenylpropanoids (more commonly known as phenolic compounds), isoprenoids, and alkaloids [19,20].

### 3.1. Phenylpropanoids

Phenylpropanoids are perhaps the most studied class of nutraceuticals in recent years [21]. Only plants, including algae, and some microorganisms are able to synthesize them from free aromatic amino acids, phenylalanine, or tyrosine [22]. The key enzyme in their biosynthesis is phenylalanine ammonia-lyase or tyrosine ammonia-lyase, responsible, respectively, for the deamination of phenylalanine and tyrosine [23].

The main classes of phenylpropanoids include stilbenes and proanthocyanidins [24]. Therefore, the term “polyphenols” is not synonymous with phenylpropanoids but identifies a specific group of phenylpropanoids with pronounced biological activities [25]. Polyphenols and other phenylpropanoids, secondary metabolites present exclusively in plant tissues, are available to humans in the form of food [26]. Their presence in fruits, vegetables, cereals, legumes, olive oil, cocoa, and plant-derived beverages (such as tea, wine, coffee, and beer) has been extensively documented [27] (Table 1). The typical Western diet involves the ingestion of more than 1 g of polyphenols per day, despite fruits and vegetables containing hundreds of other bioactive phytochemical components, in addition to these, which can exert health effects independently, additively, or synergistically [28,29]. Flavonoids have a basic chemical structure represented by the flavan nucleus, i.e., a skeleton composed of 15 carbon atoms forming three rings (C6–C3–C6): two aromatic rings (A and B) connected to a heterocyclic ring (C) containing an oxygen atom [30,31]. The main classes of flavonoids (flavonols, flavanols or catechins, flavones, flavanones, isoflavones, and anthocyanidins) differ in the level of oxidation and saturation of the C ring, while the various compounds within a class vary in the substitution pattern of rings A and B [31].

Based on the correlation between a molecule’s structure and its biological activity, the antioxidant capacity increases with the number of hydroxyl groups (–OH) and conjugated double bonds (C=C and C=O) alternated with single bonds (C–C) present on the molecule itself [32]. Among stilbenes, resveratrol and related molecules stand out, whose efficacy, although demonstrated only at the preclinical level, has even reached media attention [33]. Grapes and wine are the main sources of resveratrol and related molecules [34] (Table 1). Finally, proanthocyanidins have high molecular weight resulting from the polymerization of flavonoid units (catechins or flavanols) [35].

Among phenylpropanoids, polyphenols have been extensively studied at the preclinical level for the prevention and treatment of the most common dental pathologies (caries, periodontal disease, and candidiasis), as well as for oral mucosal cancerous and precancerous lesions. However, current data suggest a general lack of scientific evidence regarding their efficacy, with a shortage of randomized clinical trials [36].

On the other hand, epidemiological studies [37] over time have highlighted the inverse relationship between the consumption of foods rich in flavonoids and the development of oral cancer, supported by numerous preclinical studies [36,38]. Specifically, the consumption of flavonoids has been inversely correlated with the risk of oral cancer in two case-control studies, one conducted in Uruguay [38] and the other in Italy [39], with relative risks of 0.8 and 0.56, respectively [39,40]. The Italian study showed a significant inverse association, not only for total flavonoids but also for certain classes, namely flavanones and flavonols [39].

**Table 1:** The main polyphenols present in the most common foods and beverages.

Polyphenols	Foods and Beverages	Reference
Apigenin	Fruit and vegetables	[41]
Ellagic acid	Fruit and vegetables	[42]
Hesperidin	Citrus fruit	[43]
	Peppermint	[44]
Kaempferol	Green leafy vegetables	[45]
	Red wine	[46]
	Black tea	[47]
Naringenin	Orange juice	[48]
	Grapefruit juice	[49]
	Rosemary	[50]
	Red wine	[51]
Oleuropein	Olives	[52]
Quercetin	Fruit and vegetables	[53]
Resveratrol	Grapes	[54]
	Red wine	[55]

### 3.2. Isoprenoids

Isoprenoids represent a complex and diversified group of lipophilic substances widely distributed in the plant world [56]. They originate from acetyl-coenzyme A, the precursor of fatty acid biosynthesis, through the intermediate mevalonic acid [57]. Of particular interest are monoterpenes (with 10 carbon atoms) and sesquiterpenes (C15), both hydrocarbon and oxygenated, which are the main components of essential oils; triterpenes (C30), which include sterols and their steroid derivatives; and finally, tetraterpenes (C40), which include carotenoids [58].

Essential oils, in particular, are highly volatile compounds with a low threshold of olfactory perception, extracted by hydrodistillation from aromatic plants (such as mint, lavender, sage, rosemary, thyme, juniper, cypress, basil, parsley, clove, cinnamon, eucalyptus, marjoram, verbena, myrtle, sandalwood, bergamot, lemon, cumin, and anise, just to name a few) and with a high transcutaneous absorption capacity [59,60,61] (Table 2).

Their pronounced antimicrobial activity, both biocidal and biostatic, against Gram-positive and Gram-negative bacteria as well as fungi, makes them particularly useful in dentistry in the form of mouthwash [62,63]. The effectiveness of essential oils in improving plaque and gingival inflammation indices has indeed been verified, and long-term maintenance has been done as well [64].

As previously mentioned, studies published so far have demonstrated certain usefulness of mouthwashes based on essential oils in improving indicators of gingival inflammation after 6 months of treatment. Indeed, a meta-analysis study revealed that essential oils improved the gingival index and reduced plaque indices in a statistically significant manner compared to placebo. Such effects were similar to the use of chlorhexidine, although no study provided data on the incidence of gingivitis development. Furthermore, it has also highlighted that the most common side effects related to the use of such mouthwashes were increased tartar deposits and dental pigmentation [65,66].

**Table 2:** The main terpenes present in some essential oils from aromatic plants.

Terpenes	Plant Species	Common Plant Names	Reference
Capsidiol	*Capsicum annuum* L.	Red pepper	[67]
Linalool Camphor Geraniol	*Coriandrum sativum* L.	Coriander	[68]
Fenchone Limonene	*Foeniculum vulgare* Mill.	Fennel	[69]
α-pinene β-pinene Sabinene	*Laurus nobilis* L.	Laurel	[70]
Menthol Menthone Limonene Piperitone Carvone	*Mentha piperita* L.	Peppermint	[71]
Linalool Eugenol Eucalyptol	*Ocimum basilicum* L.	Basil	[72]
Thymol Carvacrol	*Origanum vulgare* L.	Oregano	[73]
1,3,8-p-menthatriene β-phellandrene	*Petroselinum crispum* Mill.	Parsley	[74]
α-pinene	*Salvia rosmarinus* L.	Rosemary	[75]
Thymol Carvacrol	*Thymus vulgaris* L.	Thyme	[76]

### 3.3. Alkaloids

Alkaloids are quaternary compounds that, unlike phenylpropanoids and isoprenoids, contain nitrogen in addition to carbon, hydrogen, and oxygen [77]. Therefore, the different groups of alkaloids each derive from an amino acid precursor, with the amino group being preserved [78].

These secondary metabolites are of considerable pharmacological interest. Some examples include atropine and cocaine, derived from ornithine [79]; morphine, derived from tyrosine [80]; catharanthine, vindoline, and camptothecin, potent anticancer agents derived from tryptophan [81,82]; and ephedrine, derived from phenylalanine [83]. In addition to these, purine alkaloids, derived from the purine nitrogenous base, are of particular interest from a nutritional point of view due to their widespread presence in the diet [84]. Caffeine, theophylline, and theobromine, found respectively in tea, coffee, and cocoa, are the main alkaloids of this family (Table 3) [85].

The rhizome (underground stem) of *Sanguinaria canadensis*, or bloodroot, contains up to 9% benzylisoquinoline alkaloids (namely sanguinarine, allocryptopine, berberine, coptisine, protopine, and stylopine). Extracts of this root have been added to toothpaste and mouthwashes, quickly gaining worldwide popularity [86].

Despite the publication of preclinical studies attesting to the antibacterial, anti-inflammatory, and antioxidant effects of this extract, its clinical efficacy against dental plaque formation and gingivitis development has not yet been clearly established. Furthermore, a concerning side effect has been highlighted, namely the potential correlation between long-term use of bloodroot in mouthwash form and the onset of leukoplakic oral lesions, histologically characterized by epithelial atrophy and hyperkeratosis [40,86,87]. In 2001, bloodroot was removed from oral hygiene products commercially available [40].

**Table 3:** The main alkaloids present in tea, coffee, and cocoa.

Alkaloids	Plant Species	Common Plant Names	References
Caffeine	*Camellia sinensis* L.	Tea	[88,89,90,91,92,93]
Theophylline	*Coffea arabica* L.	Coffee	[94,95,96,97,98,99]
Theobromine	*Theobroma cacao* L.	Cocoa	[100,101,102,103]

## 4. Nutraceutical Agents in Dentistry: Biological Effects

### 4.1. Antimicrobial and Antiviral Activity against Oral Pathogens

These compounds have various biochemical and molecular targets against bacterial and fungal metabolism, also inhibiting viral replication [104]. In particular, an interesting study proved the antibacterial and antibiofilm activity of honokiol and magnolol, two natural components isolated from *Magnolia* bark, an officinal plant used in traditional Chinese and Japanese herbal medicine. In this work, the authors revealed that honokiol more efficiently inhibited the growth and the biofilm formation of oral bacteria compared to magnolol. Moreover, both compounds were able to reduce the expression of pro-inflammatory genes in the human macrophage RAW26bb4.7 cell line [105]. A plethora of in vitro studies showed the antibacterial and antibiofilm activity of polyphenols against different periodontal pathogens [106]. In particular, the Cranberry polyphenol fraction efficiently inhibited the biofilm formation by *Porphyromonas gingivalis* (*P. gingivalis*) and *Fusobacterium nucleatum* (*F. nucleatum*) and affected some *P. gingivalis* proteases [107]. Catechins contained in wine showed potent antimicrobial activity against *P. gingivalis* and *Prevotella intermedia* (*P. intermedia*) [108]. Green tea catechins, slowly released into gum pockets via a local delivery strip, reduced pocket depth and the number of harmful, anaerobic bacteria. These same catechins were also shown to kill *P. gingivalis* and *Prevotella* spp. [109]. A further study proved the antibacterial activity of curcumin, a polyphenol obtained from the root of *Curcuma longa* (*C. longa*), against numerous periodontopathic bacteria, including *P. gingivalis*, *P. intermedia*, *F. nucleatum*, and *Treponema denticola* [110]. Regarding alkaloids, Hu et al. demonstrated the antibacterial activity of berberine against *Aggregatibacter actinomycetemcomitans* (*A. actinomycetemcomitans)* and *P. gingivalis*, thanks to the inhibition of the collagenase enzyme [111]. In addition, different plant extracts containing high amounts of alkaloids exerted antiviral activity against the *Herpes simplex* virus and hepatitis viruses A, B, C, and D. This effect could be due to the increase of interferon production in the host [112].

### 4.2. Anti-Inflammatory Activity

It has been demonstrated that polyphenols are able to inhibit pro-inflammatory signaling pathways, such as the cyclooxygenase (COX)/lipoxygenase (LOX) system [113] and the production of proinflammatory cytokines (e.g., interleukins IL-1, IL-8) involved in some inflammatory oral diseases [114]. They also reduce osteoclastic activity by stimulating monocytes/macrophages to produce anti-inflammatory cytokines [106]. A recent study reported that participants consuming nutritional supplements displayed a reduced increase in C-reactive protein (CRP), a well-established inflammatory biomarker, compared to the control group. Furthermore, a slight beneficial effect on gingival inflammation was observed in the supplement group. Both groups were dental students undergoing comparable exam-induced stress [115]. The cranberry proanthocyanins exert anti-inflammatory activity by reducing the release of pro-inflammatory cytokines, such as IL-6, IL-8, and TNF-α from gingival fibroblasts, the cell type mainly involved in the inflammatory response in the periodontal tissues [116,117]. Furthermore, proanthocyanidin-enriched cranberry fraction exerted anti-inflammatory action by reducing COX-2 expression and the release of pro-inflammatory cytokines IL-6, IL-8, and prostaglandin E2 production from gingival fibroblasts stimulated by the lipopolysaccharide (LPS) of *A. actinomycetemcomitans* [118]. An interesting in vivo and in vitro study proved the anti-inflammatory activity of resveratrol. Indeed, the polyphenol resveratrol attenuated the inflammatory response on human gingival fibroblasts treated with LPS. Specifically, it was able to counteract the increment in the expression of COX-2, matrix metalloproteinase (MMP)-2, MMP-9, and Toll-like receptor-4 induced by LPS treatment. In addition, resveratrol was able to enhance the expression of heme oxygenase-1 (HO-1) via nuclear factor-E2 related factor 2 (Nrf2), an antioxidant pathway that was inhibited by LPS. Moreover, in periodontitis rats, resveratrol exerted anti-osteoclastogenic activity and enhanced the SOD activity in blood serum, compared with LPS-treated rats [119].

In a clinical study by Vaziri et al. [120], the authors confirmed the protective effect in aphthous ulcers of a mouthwash derived from the leaves of *Nicotiana tabacum* L. (*N. tabacum*). The main compound present in *N. tabacum* leaves is the alkaloid nicotine, to which the aforementioned effect was attributed. Nicotine can induce an immunosuppressive condition and reduce inflammation by several mechanisms, including the inhibition of the production of IL-1β, IL-2, IL-8, IL-10, TNF-α, and Interferon-γ [121,122].

### 4.3. Antitumor Activity

Similar to their antibiotic activity, nutraceutical substances can be considered “multitarget” antitumor agents, particularly favorable for controlling multifactorial diseases such as tumors. They act at the cellular cycle, apoptosis, steroid receptors, angiogenesis, and metastasis levels [123]. In this regard, naringenin, a polyphenol mainly present in citrus fruits, such as grapefruits, tomatoes, and cherries, showed anticancer activity against a large variety of human cancer cell lines, including the oral squamous carcinoma cell line. In this work, the authors demonstrated that naringenin hindered cancer cell growth through different mechanisms, including the activation of apoptotic cell death, cell cycle arrest, and angiogenesis hindrance. These effects reflect the ability of this polyphenol to modulate various signaling pathways, including Wnt/β-catenin, PI3K/AKT, NF-ĸB, and TGF-β pathways [124]. To enhance the efficacy of natural compounds, different nanostrategies were developed. Concerning that, gold nanoparticles (AuNPs) containing Punica granatum peel exerted anticancer effects against human oral squamous cell carcinoma (HSC-2, HSC-3, HSC-4, and Ca9-22) cell lines without affecting HUVEC cell viability [125]. A recent study highlighted the anticancer activity of a natural polyphenol, the thymoquinone. Specifically, the authors proved the ability of this natural compound to enhance the efficacy of the anticancer drug cisplatin. Indeed, the combination of the two agents affected human oral squamous carcinoma SCC-25 cell viability and induced apoptosis in a dose-dependent manner. Moreover, the treatment of SCC-25 cells with both natural and synthetic agents determined a significant increase in intracellular ROS levels, thus inducing DNA damage and protein and lipid peroxidation. Moreover, the two compounds synergistically modulated apoptosis-related proteins, including Bax, Bcl-2, and caspase-3 [126]. In literature, it has also been reported that several alkaloids exert a protective effect against oral cancer. Specifically, Sun et al. demonstrated that colchicine, an alkaloid extracted from the plants of the genus *Colchicum*, determined the death of oral cancer cells through the hyperproduction of cytosolic Ca^2+^ [127]. Zhang et al. studied the antitumor activity of heptazoline, a carbazole alkaloid from *Clausena* spp., against SCC-15 oral cancer cells. In particular, heptazoline induced cell apoptosis through the overexpression of caspase-3. Furthermore, this compound determined a decrease in the phosphorylation of PI3K and AKT [128].

## 5. Oral Pathologies and Nutraceutical Agents

The use of medicinal herbs and plant extracts is steadily increasing in the biomedical field, and the number of people using them for aspects of their daily care, such as oral hygiene, is growing [129]. Table 4 summarizes the clinical studies currently available regarding the use of nutraceutical substances in dentistry; each oral disease and the specific nutraceutical agents are detailed in the following paragraphs. The rationale behind the use of nutraceutical substances in dentistry lies in their ability to perform three main actions, upon which their preventive and therapeutic effects are based.

### 5.1. Dental Caries

Dental plaque, a bacterial biofilm adhering to dental and mucosal surfaces, is considered the etiological agent of caries [165]. *Streptococcus mutans* is the main bacterium involved: it can adhere to the dental substrate through specific proteins, while lactic acid, produced by the fermentation of dietary sucrose via the enzyme glucosyltransferase, causes enamel demineralization, leading to cavity formation [166]. A recent review suggested that polyphenols inhibit the proliferation of cariogenic bacteria, their adhesion, and co-aggregation on enamel and dentin, also reducing the activity of glucosyltransferase and salivary amylases [167]. In humans, a reduction in bacterial adhesion to dental surfaces was observed after rinsing with 200 mL of grape juice [130], and a reduction in plaque after rinsing with oolong tea extract (0.5 mg/mL) [168]. An epidemiological study also reported a decrease in caries indices in 14-year-old English children who habitually drank tea [169]. *Theobroma cacao* (Cocoa) has also been studied for its anticariogenic effects. Cocoa beans, in particular, have been used to produce an anticaries mouthwash for children, whose regular use resulted in a reduction of *S. mutans* contamination and an improvement in plaque index [131]. However, the anticariogenic effect of cocoa components remains controversial: a high-sugar diet is equally cariogenic, both in the presence and absence of cocoa [170], and there were no differences between chocolates with different percentages of cocoa [171]. The extract of magnolia bark (*Magnolia grandiflora*), administered daily as sugar-free chewing gum, was able to prevent the development of caries and gingivitis [132]. An Italian study on 120 highly caries-susceptible adult volunteers compared this product to a placebo and a chewing gum containing xylitol: the magnolia chewing gum significantly reduced plaque acidogenicity, the salivary concentration of *S. mutans*, and gingival bleeding compared to xylitol and control after 30 days of use [132]. Contradictory results on plaque formation have been reported with the use of toothpaste containing different concentrations of glycyrrhizin, an isoprenoid extracted from licorice, *Glycyrrhiza glabra* [172,173]. Sugar-free licorice lollipops, in conclusion, represent a very appealing perspective against caries pathology, especially in pediatric age. In two studies, their use produced a reduction in salivary concentration of *S. mutans* [174,175]. However, one must not forget the main adverse effect associated with excessive consumption of the product, namely arterial hypertension [176]. The studies conducted so far are not associated with a certain clinical impact in preventing caries pathology through the aforementioned natural products, considering the limited sample sizes and the assessment of secondary indicators such as plaque index and microbiological tests [133].

### 5.2. Periodontal Diseases

Periodontal diseases are due to inflammatory processes of the periodontal apparatus, in turn, caused by bacteria present in the supragingival and subgingival plaques [177]. The main periodontopathogens identified are *P. gingivalis* [178], *Streptococcus sanguinis* [179], and *Aggregatibacter actinomycetemcomitans* [180]. Dental plaque and tartar, along with smoking, genetic predisposition, and the presence of certain systemic diseases, contribute to the development of periodontitis [181]. Previous studies, reporting a reduction in bacterial plaque following rinsing with grape juice [130] or oolong tea extract [168], suggest a preventive effect of these products against plaque-related periodontal disease. A recent epidemiological study [182] also found a slight inverse association between green tea intake and the development of periodontal disease, with a reduction in pocket depth, clinical attachment loss, and bleeding on probing. The reported values have, in this case as well, questionable clinical relevance, although reaching statistical significance (*p* < 0.05): after adjustment for confounding factors, one cup of green tea per day was associated with a decrease in probing depth of 0.023 mm and a reduction in attachment loss of 0.028 mm, with a decrease in bleeding percentage by 0.63. Finally, an *Aloe vera* (*A. vera*)-based toothpaste has been studied for its supposed ability to reduce plaque and gingival inflammation without finding any difference compared to a fluoride-containing toothpaste [183]. A study verified the antibacterial effect of propolis (a bee product rich in essential oils and phenolic compounds) on saliva samples from healthy subjects and patients with chronic periodontitis. The antibacterial action was evident, although to a lesser extent than the activity of chlorhexidine [134], considered the gold standard antiseptic agent in dentistry against periodontopathogenic bacteria [65,66]. In comparison to chlorhexidine, a curcuma-based mouthwash (*C. longa*) showed equal efficacy in reducing gingival index but lower efficacy as an anti-plaque agent [135]. A new fluoride toothpaste based on Himalayan herbs was recently tested in a cohort of young women without finding any differences compared to a common commercial fluoride toothpaste in reducing gingival and plaque indices [184]. Consistent with these studies, a mouthwash based on Himalayan herbs (*Tinospora cordifolia*, *Commiphora wightii*, and licorice), proposed as an immunomodulatory in the treatment of periodontal disease, was found to be entirely ineffective; moreover, when taken in tablet form for 2 weeks after causal periodontal therapy, it did not result in significant improvements in periodontal indices compared to mechanical causal therapy alone [185]. Three other mouthwashes—one based on the fruits of *Terminalia chebula*, *Terminalia bellirica*, and *Phyllanthus emblica*, plants used in traditional Ayurvedic medicine; a second Ayurvedic formulation based on *Syzygium aromaticum*, *Cinnamomum zeylanicum*, *Spinacia oleracea*, *Piper nigrum*, and *Zingiber officinalis*; a third mouthwash based on *Centella asiatica*, *Echinacea purpurea*, and *Sambucus nigra*—were tested in a double-blind, crossover, randomized study in healthy subjects, with preliminary results [186]: the first two mouthwashes reduced new plaque formation, at 24 h, similarly to chlorhexidine rinses, but more effectively than a commercial mouthwash (*p* < 0.05); the third was associated with lower gingival inflammation indices compared to mouthwashes containing essential oils or cetylpyridinium chloride; the third one was associated with lower gingival inflammation indices compared to mouthwashes based on essential oils or cetylpyridinium chloride; a clinical study combined normal mechanical therapy with the local application of green tea catechins using slow-release oral strips. The results were promising, despite the small number of enrolled subjects (6 periodontopathic volunteers) [109]. A second type of transmucosal local release (patch) was impregnated with plant extracts (based on *Centella asiatica*, *Echinacea purpurea*, and *Sambucus nigra*) and used to evaluate the reduction of gingival inflammation in patients with gingivitis [187]: the placebo-controlled, randomized, phase II study on 53 patients showed some efficacy in reducing the gingival inflammation index. Local release systems of nutraceutical substances undoubtedly represent the future, aiming to enhance their bioavailability and pharmacological activity, although the results obtained so far need to be confirmed with larger and more in-depth studies.

### 5.3. Oral Mucosal Lesions

Due to their anti-inflammatory activity, a preventive and/or therapeutic action of nutraceutical substances against different pathological conditions has been hypothesized. Specifically, they could be useful in the treatment of some autoimmune-based oral mucosa diseases, including recurrent aphthous stomatitis, oral lichen planus, and mucous membrane pemphigoid. Moreover, natural agents could exert a protective action against infectious diseases (e.g., candidiasis) or head and neck radiotherapy-induced mucositis [188]. Some nutraceutical agents have also been proposed in the chemoprevention and treatment of oral carcinoma, based on studies supporting their antiproliferative and proapoptotic action [123]. Recurrent aphthous stomatitis is characterized by the presence of painful ulcers of the oral mucosa [189]. It is the most common ulcerative oral disease in developed countries, affecting one-third of the world’s population in its milder form [190]. The etiology remains unknown, while delayed hypersensitivity has been suggested as a key element in its pathogenesis [191]. The aphthous ulcers, of variable diameter (1–10 mm), are recurrent, moderately or very painful, with circumscribed margins, and usually involve non-keratinized mucosa (margins and ventral tongue). They last 4–14 days, and their onset seems to be correlated with many exacerbating factors, such as trauma, stress, and hormonal changes. Their treatment remains highly controversial [192].

A group of Japanese researchers conducted, in 2006, an intervention study to prevent minor aphthous stomatitis [136]. The randomized, double-blind, placebo-controlled study aimed to evaluate the effect of using *Perilla frutescens* oil (rich in alpha-linolenic acid) as a cooking ingredient for 8 months in 30 patients, comparing it with a 50:50 mixture of soybean oil and rapeseed oil. The study results did not show differences in terms of the frequency of the condition between the two groups; however, a reduction in prevalence was reported in both cases. A clinical study examined the effectiveness and safety of a chamomile extract in treating pain associated with aphthous ulcers: the analgesic effect, measured using the visual analog scale, was considered excellent in 82% of patients, albeit short-lived [137]. In a randomized, double-blind clinical study involving 45 patients with recurrent aphthous stomatitis, the use of a new myrtle-based paste (*Myrtus communis*) was also proposed [138]. The subjects were treated with either a placebo or myrtle paste, applied four times a day for 6 days, in two consecutive episodes of aphthae. The results indicate a statistically significant reduction in the size of ulcers, pain severity, erythema, and exudate in the group treated with myrtle. The oral health impact profile in these subjects improved significantly, suggesting that the myrtle-based product may be effective in improving the quality of life in patients suffering from this disorder [138]. Among the most widely used commercial products in recent years for the treatment of aphthous lesions are *A. vera*-based gels or adhesive disks, known for their well-established healing properties [193]. However, the effectiveness of *A. vera* gel in promoting the healing of recurrent aphthous stomatitis ulcers has been evaluated by only one clinical study, divided into multiple phases, involving subjects with recurrent aphthous lesions every 3 or 4 months [139]. The gel was in combination with two other active ingredients (allantoin and silica dioxide). The results indicate a level of effectiveness considered acceptable by the authors only when all three active ingredients were present. In this condition, they showed the ability to reduce the number of lesions and increase the duration of lesion-free periods. However, no direct effect of the gel in promoting the healing of aphthous ulcers was highlighted.

Recently, preliminary clinical studies have shown an effect of licorice in reducing pain and healing time of ulcers, particularly in the form of mouthwash [194] and oral patches [140,195]. A distillate of *Acacia erioloba* has slightly reduced the healing time of aphthous ulcers; the authors suggest a probable action linked to the presence of flavanones (alhagitin and alhagidin) [141].

### 5.4. Oral Candidiasis

Oral candidiasis is an inflammatory disease caused by the proliferation of *Candida albicans* (*C. albicans*), a commensal ascomycete fungus of mucous membranes [196]. *C. albicans* is the most frequently isolated yeast, although an increasing number of other *Candida* species have been reported, especially in immunocompromised patients [197]. A peculiar characteristic of this fungus lies in its dimorphism, i.e., its ability to transition, under certain environmental conditions, within the human body from a yeast-like growth form (with spherical cells) to a filamentous form, thereby enhancing its virulence [198]. Clinically, oral candidiasis can vary from the form with white pseudomembranes on mucous membranes to atrophic and erythematous forms to the rarer hyperplastic forms [199]. One of the most common forms is denture stomatitis: erythematous candidiasis “stamped” under the prosthetic base [200].

The standard treatment for denture stomatitis involves the topical use of antifungal drugs, such as nystatin [201]. Although not associated with significant adverse effects and demonstrating efficacy, nystatin has a bitter taste and, albeit rarely, can interact with statins, causing myositis, or induce resistance phenomena [202]. To find alternative antifungal compounds, the efficacy of the most common antifungals used for denture stomatitis treatment has been compared with natural extracts. Specifically, a randomized Iranian clinical study of 40 patients evaluated the effect of Garlic (Allium sativum). The results revealed that Garlic use for 4 weeks decreased the degree of erythema, albeit more slowly than nystatin. Nevertheless, overall the results generated great “satisfaction”, as the extract was better accepted than the drug [142]. A further study, involving 30 elderly subjects, evaluated the efficacy of castor oil (*Ricinus communis*) compared with nystatin and miconazole. Evaluating both clinical and microbiological responses through salivary swabs for fungal colony counts, the results obtained show a certain effect of castor oil, comparable to miconazole, in reducing the clinical picture of stomatitis, although it does not affect microbiological counts [143].

Interesting is a study, albeit dated, that evaluated the efficacy of an oral suspension of *Melaleuca alternifolia* oil in the treatment of oral candidiasis resistant to fluconazole in HIV/AIDS patients: 60% of patients showed a clinically significant improvement after 4 weeks of treatment [144]. Other products, evaluated through randomized controlled clinical trials, albeit on small samples, include gels based on 0.1% *Zataria multiflora*, applied 4 times a day for 14 days [145]; and *P. granatum*, administered 3 times a day for 15 days. Both showed similar clinical outcomes to miconazole gel treatment [146].

### 5.5. Oral Lichen Planus

Oral Lichen Planus (OLP) is a chronic inflammatory disease of the oral cavity, caused by an immune reaction, mostly lymphocytic, to an unknown antigen localized at the level of the basal layer of the stratified squamous epithelium of the oral mucosa [203]. The action of CD8+ lymphocytes leads to the apoptosis of epithelial cells and the activation of metalloproteinases, causing damage to the epithelial membranes [203]. This results in the typical clinical lesions, represented by white, reticular striae, bilateral on the buccal mucosa, with or without areas of erosion or ulcers, often associated with intense burning sensation [204].

*A. vera* has been proposed for the treatment of OLP based on evidence related to its aforementioned anti-inflammatory, immunomodulatory, and wound-healing properties [205]. Two randomized controlled clinical studies have been conducted to evaluate its efficacy, although corticosteroid-based therapy, currently the reference treatment, was not considered in either case [147,148]. The first study, conducted on 64 patients in a double-blind fashion, involved the topical application of a 70% *A. vera* extract three times a day for 12 weeks. The difference between *A. vera* and placebo in terms of pain reduction at 6 and 12 weeks was not statistically significant, although topical application of *A. vera* seemed to improve the overall quality of life of patients [147].

In the second study, involving a sample of 54 patients divided into two groups treated with *A. vera* gel or placebo for 8 weeks, the former treatment was significantly more effective than the latter in inducing improvement in terms of symptoms and clinical signs [148].

Both groups reported no side effects. Their findings are consistent with a meta-analysis study by the Cochrane Collaboration [206]. Treatment of OLP with *A. vera* appears to be effective in reducing pain. Chainani-Wu et al. [207] evaluated the efficacy of a 95% curcuminoid extract (flavonoids extracted from *C. longa*) administered at 2000 mg/day for 7 weeks in the treatment of OLP in a randomized, double-blind, placebo-controlled study. Unfortunately, all subjects had received prednisone (60 mg/day) for the first week, which was a substantial confounding factor. Nevertheless, the administration of curcuminoids showed good compliance and was well-tolerated by patients, and the frequency of side effects was similar in both groups. There was no difference between placebo and extract in terms of symptom and clinical sign improvement, and the study was prematurely terminated due to this lack of efficacy. In conclusion, there is no clinical evidence of the efficacy of curcuminoids in OLP.

### 5.6. Oral Leukoplakia

Oral Leukoplakia is defined as “a white patch that cannot be clinically and histologically characterized as any other oral disease” [208]. It is a potentially malignant lesion, usually painless and localized on the ventral and/or lateral borders of the tongue and on the attached gums; it can be homogeneous, heterogeneous, or verrucous, with sharp or undefined margins [208].

The etiopathogenesis is unknown, but evidence suggests that the risk of contracting this disease is much higher in smokers and betel nut chewers [209]. Histologically, leukoplakia can range from simple epithelial hyperplasia to various degrees of dysplasia [209]. At present, no clinical or histological findings can predict which of these lesions will evolve into cancer [209]. There is currently no unanimous agreement on the best therapeutic approach, although close patient follow-up remains crucial, given the precancerous nature of the lesion [210].

A randomized double-blind study, conducted on 59 patients with oral leukoplakia, compared the oral administration, in capsule form, of 3 g/day of tea extract along with topical administration, in the form of a 10% ointment, of the same extract (treated group) with the administration of the same capsules along with a glycerin-based placebo (control group) [149]. The topical application of the ointment aimed to increase the local effect of the product directly on the lesions. After 6 months of treatment, the size of oral lesions decreased in 37.9% of the subjects in the treated group and increased in 3.4% of the 29 patients; in the control group, instead, lesions decreased in 10.0% and increased in 6.7% of the 30 patients. Another phase II randomized, placebo-controlled study on 41 patients evaluated the effect of green tea extract in the treatment of leukoplakic lesions considered at high risk of malignant transformation. Patients were randomly assigned to receive green tea extract 1.0 g/m^2^ (n = 10) or 0.75 g/m^2^ (n = 9) or 0.5 g/m^2^ (n = 11) or placebo, three times a day for 12 weeks [150].

The effectiveness was determined by assessing the disappearance of all lesions (complete response) or the percentage decrease in their size (partial response). Although not reaching statistical significance, the two arms receiving the highest concentrations of extract (0.75 and 1.0 g/m^2^) had a higher response rate (58.8% reduction in lesions) compared to those receiving the extract at 0.5 g/m^2^ (36.4%) or placebo (18.2%). Among the side effects, present at the highest concentration, there was insomnia, probably due to the caffeine content as an excipient in the extract, diarrhea, and oro-facial pain. At the 27.5-month follow-up, there were no differences between the groups in terms of survival without oral carcinoma. In addition to green tea, black tea has also been investigated. A preliminary study in patients with oral leukoplakia showed some clinical improvement with a decrease in the frequency of micronuclei and chromosomal aberrations [211].

However, it is unclear whether tea extracts are able to reduce the frequency of evolution into cancer. A study proposed the local use of a gel based on blackberry in the treatment of oral leukoplakia lesions and their prevention in terms of tumor transformation [151].

The gel was observed to suppress the expression profile of genes related to intraepithelial oral neoplasia, involved in RNA processing, recycling of growth factors, and apoptosis inhibition [151]. The treatment also reduced, at the level of superficial connective tissues, the epithelial COX-2 protein, vascularization density, and genes associated with terminal keratinocyte differentiation [151]. In the context of a large study [152] aimed at evaluating the efficacy of curcumin (extracted from the root of *C. longa*) in reducing oncological risk in different pathological conditions, oral leukoplakia was also considered. Curcumin was orally administered for 3 months. From biopsies taken immediately before and after 3 months of treatment, it emerged that 1 out of 7 patients had developed oral cancer, while 2 out of 7 had shown histological improvement of the lesion [152]. However, it is not possible to draw definite conclusions from this study, given the small sample size and the short follow-up period considered.

Finally, in a clinical study published by a group of Chinese researchers, a mixture of herbs based on *Sophora tonkinensis*, *Polygonum bistorta*, *Prunella vulgaris*, *Sonchus brachyotus*, *Dictamnus dasycarpus*, and *Dioscorea bulbifera* was used in patients with oral leukoplakia (4 tablets 3 times a day for 8–12 weeks) [153]. The mixture appeared to reduce not only the expression of two tissue biomarkers but also the size of oral lesions in 67.8% of cases (40 out of 59 patients) compared to 17% in the placebo group (9 out of 53 patients).

### 5.7. Oral Carcinoma

Oral carcinoma accounts for 90% of all tumors in the oral cavity and is the sixth most common tumor worldwide, with very low survival if diagnosed late [212]. It does not present a single clinical form; more often it appears as a constantly growing mass, sometimes ulcerated, with an irregular surface and white-red color [212].

Multiple preventive campaigns, supported by the World Health Organization, have been directed towards promoting healthy lifestyles (adequate consumption of fruits and vegetables) and against habits that promote its development (cigarette smoking and alcohol abuse). Smoking alone represents the main environmental factor responsible for oral carcinogenesis. If alcohol abuse is also associated with smoking (20 cigarettes a day for over 20 years), the risk increases by 40 times. The carcinogenic or potentially carcinogenic substances present in cigarette smoke are numerous, and their absorption through the oral mucosa is considerably favored by ethanol, capable of permeabilizing the epithelium.

Oxidative stress generated by tobacco smoke, along with genetic predisposition, can cause genomic instability in the oral mucosa, predisposing to tumor development through different molecular mechanisms [213]. The most important of these is the inhibition of the Tumor Growth Factor (TGF), which regulates genes suppressing cell proliferation; loss of the TGF-related signal is associated with increased proliferation and cell survival [214]. The antioxidant, antiproliferative, and proapoptotic properties of nutraceutical substances abundant in plant-derived foods have been associated with the mechanism of chemoprevention, potentially able to protect individuals from the carcinogenesis process [123]. Despite epidemiological evidence suggesting an association between a diet rich in nutraceutical substances and a reduced incidence of oral cancer [215], the mechanisms by which these phytochemical compounds exert their anti-tumor action remain not completely understood. The main protective mechanisms, in particular, are as follows: direct antioxidant action of nutraceuticals and their ability to stimulate the cell’s endogenous antioxidant defences, both enzymatic and non-enzymatic; inhibition of enzymes involved in oxidative stress, such as nitric oxide synthase, peroxidases, LOX, COX, and xanthine oxidase; induction of apoptosis in tumor cells; inhibition of binding between DNA and carcinogens; inhibition of cyclin-dependent kinases [106,123,188].

As already reported, epidemiological data have suggested the preventive effect of diets rich in fruits and vegetables [216]. A meta-analysis of case-control studies has also found an inverse association between coffee consumption and the risk of oral cancer [217]. A similar conclusion was reported in a recent meta-analysis of observational studies, both case-control and cohort studies [218]. On the other hand, tea consumption is among the most investigated aspects of reducing the risk of oral cancer [219]. The main antioxidants present in green tea are catechins (particularly epigallocatechin gallate, EGCG; Table 1), while in black tea, theaflavins and thearubigins formed by oxidation and polymerization of catechins during fermentation are found [220]. Presumably, the two groups of compounds possess different biological activities. A prospective study conducted in Japan [221] involved a total of 20,550 men and 29,671 women aged 40 to 79 years without a history of oral carcinoma. A mean follow-up period of about 10.3 years identified 37 cases of oral carcinoma. For women, the tumor risk was 0.51, 0.60, and 0.31 depending on the number of cups of green tea consumed daily (1–2, 3–4, or 5, respectively) compared to those who consumed only one cup per day. For men, the inverse association was slightly lower and, in any case, did not reach statistical significance due to the small number of carcinoma cases detected. A second prospective cohort study, initiated in 1982, involving 968,432 men and women with no history of tumors, failed to correlate tea (black or green) consumption with a reduced risk of oral cancer [222].

On the contrary, the ICARE study (Investigation of Occupational and Environmental Causes of Respiratory Cancers), a large multicentric population-based case-control study involving subjects with lung and head and neck cancer, conducted between 2001 and 2007 in France, revealed an association between oral cavity carcinoma and tea or coffee intake [222]. Finally, according to a meta-analysis of 51 studies involving more than 1.6 million participants conducted by the Cochrane Collaboration, the evidence that green tea consumption may reduce the risk of oral cancer is controversial and not yet established [223]. Intervention studies, concerning chemoprevention, also refer to the usefulness of nutraceutical compounds in the therapy of oral premalignant lesions (leukoplakia), considered risk factors for oral carcinoma, as mentioned above [224]. However, to date, the positive effect of using such compounds in reducing the frequency of progression of precancerous lesions to carcinoma, which remains the primary outcome of such treatments, is still unclear [225].

Few studies have actually evaluated this aspect, mostly focusing on “indirect” indicators such as the improvement of tumor biomarkers and histological features. In an attempt to understand the protective mechanisms involved in chemopreventive effects in at-risk individuals, green tea extracts, rich in catechins, have been the subject of clinical investigation in heavy smokers [154].

Therefore, at risk of oral precancerous and cancerous lesions, the study evaluated the capacity of a green tea extract to reduce DNA damage in oral keratinocytes: the extract, administered at a concentration of 2000–2500 mg/day for 4 weeks, inhibited cell growth, increased the content of diploid DNA, and decreased that of aneuploid DNA, while apoptosis markers were found to be overexpressed [154].

In a study on patients with previous oral carcinoma or a clinical history of recurrent dysplastic lesions, the topical application of EGCG in the form of rinses for 7 days reduced the expression levels of some biomarkers of oral carcinogenesis, although without statistical significance [226]. Significant amounts of EGCG were found in saliva but not in plasma, demonstrating a certain local bioavailability of the catechin in the absence of significant systemic absorption [226].

Recently, blackberry extracts, high in anthocyanins, have also been proposed for topical application in gel form. In healthy volunteers, application on the adherent mandibular gum produced measurable levels of these flavonoids in saliva and oral tissues, demonstrating how anthocyanins are rapidly released into saliva, easily penetrating the oral mucosa [227].

In a small clinical study, 17 patients with premalignant lesions were evaluated through pre- and post-treatment biopsies and by determining loss of heterozygosity, a secondary indicator of lesion severity. Seven lesions showed histological improvement, six remained stable, and four worsened [155].

The studies reported so far do not allow us to ascertain a clear chemopreventive effect of individual compounds or plant extracts. It is possible that the protective link identified through epidemiological studies takes into account a global effect related to overall dietary and nutritional factors and that this effect, based on synergy among different nutraceutical agents, may somehow act as a protective factor against oral cancer.

Furthermore, one of the major controversies regarding the limited action of many natural origin nutraceutical chemo-preventive agents concerns their limited bioavailability. Consequently, despite promising results from preclinical studies, the use of such substances in humans has only yielded limited effectiveness. Recently, two new approaches have been proposed to improve bioavailability. The first, based on nanotechnology, relies on the concept of nanochemoprevention, which involves encapsulating natural agents in biocompatible nanoparticles to enhance their stability and absorption [123]. The evidence available to date regarding the use of nanobiomaterials for releasing such compounds, although promising, remains only at the preclinical level.

The second approach involves the topical application of these compounds, exploiting the transmucosal route and following two possible transport pathways: paracellular (through intercellular spaces, generally preferred by hydrophilic compounds) or transcellular (through cell membranes and cytoplasm, preferred by lipophilic substances). The transmucosal route offers many advantages over oral or systemic applications. The oral mucosa is, in some sites, poorly keratinized and highly vascularized, making it well-suited for the absorption of locally applied agents and avoiding first-pass hepatic metabolism and presystemic elimination within the gastrointestinal tract. Buccal and/or sublingual release systems include mouthwashes, aerosol sprays, chewing gum, bioadhesive disks, and gels [123].

### 5.8. Oral Mucositis

Oral mucositis is an acute and severe inflammation of the oral mucosa that affects patients undergoing immunosuppressive therapy (methotrexate or 5-fluorouracil) or exposed to head and neck radiotherapy, manifesting as progressive damage to the epithelial layers due to antitumor therapy. It represents one of the most important side effects of these oncological therapies, linked to their non-selective cytotoxicity, primarily directed against cells with a high rate of replication, such as tumor and epithelial cells [228]. The pathogenesis of oral mucositis consists of widespread damage to the oral mucosal epithelium, combined with an inhibition of the immune system, resulting in a very painful condition with oral ulcers and atrophic and erythematous lesions [229]. Current therapies are purely palliative, and their clinical value has not yet been demonstrated [230].

A review by the Cochrane Collaboration [231] proposed an *A. vera*-based toothpaste as a potential intervention for the prevention of oral mucositis in patients undergoing systemic anticancer therapies; although without a definitive conclusion on its efficacy, this product appears to reduce the severity of the disease.

A clinical case of oral mucositis induced by methotrexate in a patient with rheumatoid arthritis was successfully treated using chamomile mouthwashes [156], while another prospective phase III clinical study, double-blind and placebo-controlled, reported discouraging results [157]. In this latter study, a mouthwash based on chamomile extract was tested on cases of oral mucositis induced by 5-fluorouracil. Specifically, 164 patients received oral cryotherapy for 30 min with each dose of 5-fluorouracil. Then they were randomized to receive either chamomile extract or placebo as mouthwashes, three times a day for two weeks. The results did not show any differences between chamomile and placebo [157].

A recent single-blind, placebo-controlled study evaluated the safety and efficacy of a formulation based on plant extracts of *Vaccinium myrtillus*, *Macleaya cordata*, and *Echinacea angustifolia* in the treatment of oral mucositis, administered during chemo/radiotherapy, four times a day for 50 days [158]. With negligible adverse effects, patients treated with the plant extracts (n = 20) reported subjective improvement in pain and quality of life, with greater ease in eating, drinking, and speaking compared to the placebo group (n = 10).

Finally, based on observational studies, a compound called HOPE has recently been proposed, which defines an ointment composed of honey, olive oil, propolis, and beeswax. Tested on 90 patients with moderate to severe mucositis in a controlled and randomized clinical trial, faster healing was reported, offering prospects for further studies [159].

### 5.9. Burning Mouth Syndrome

Burning Mouth Syndrome (BMS) is a condition associated with an intense sensation of burning or stinging pain, mainly localized at the tip of the tongue, in the absence of evident pathological clinical signs upon examination of the oral mucosa and laboratory test abnormalities [232]. To date, there is no effective treatment, despite numerous trials using antidepressant drugs and natural substances [160].

A recent review [160] indicated that local treatment with alpha-lipoic acid or capsaicin, as well as clonazepam, was associated with a reduction in symptoms in patients with BMS, although complete remission was not achieved [161]. Recently, a randomized, double-blind, placebo-controlled study tested the efficacy of a plant-based preparation consisting of *Paullinia cupana* (guarana), *Trichilia catigua*, *Zingiber officinalis* (ginger), and *Ptychopetalum olacoides* (2 capsules per day for 8 weeks) [162]: both groups showed a reduction in symptoms, but the treated group reported a significantly greater improvement compared to the placebo, especially at 8 weeks. The systemic administration of the preparation appears to reduce the symptoms associated with BMS, although further confirmation is needed.

### 5.10. Oral Submucous Fibrosis

It is a precancerous condition associated with the chewing of betel nut (*Areca catechu*), which contains substances capable of causing a local condition of oxidative stress resulting in damage to oral tissues [233]. Rare in Italy, it is commonly found in Southeast Asia, where the habit of chewing betel nuts is widespread [233]. To date, there is no effective treatment [233].

Salvianolic acid has been tested in a randomized clinical trial in patients with oral submucous fibrosis. Used in the form of intralesional infiltration once a week for 20 weeks and associated with treatment with triamcinolone acetonide, an improvement in mouth opening and burning sensation was observed [163]. Similarly, a study on 20 patients compared the effect of applying an *A. vera* gel (5 mg 3 times a day) with that of some vitamin antioxidants [164]. Good safety of use, low cost, and ease of application were highlighted, as well as a better efficacy in reducing the burning sensation and promoting mouth opening compared to antioxidant agents. However, the studies, few and on a small number of patients, do not provide reliable evidence of efficacy, although they suggest a clinical potential, especially regarding the use of *A. vera* gel, worthy of further investigation with larger studies.

## 6. Granted Patents for Oral Care Products Containing Natural Compounds

The multiple biological properties, including the antimicrobial, anti-inflammatory, and antioxidant activities of natural compounds, made them valuable candidates for the development of new oral care products able to prevent or treat oral diseases. These natural compounds offer promising alternatives or adjuncts to conventional treatments for oral diseases, particularly in reducing the reliance on synthetic chemicals and antibiotics. Considering that a large number of patents have been granted, some of them are listed in Table 5.

## 7. Nutritional and Behavioral Preventive Recommendations

The nutritional and behavioral preventive recommendations that dentists are called upon to provide to their adult patients are as follows [244,245,246]: eat fruits and vegetables (preferably seasonal) and legumes and whole grains (bread, pasta, rice, and breakfast cereals); dress salads with extra virgin olive oil, also used in (few) fryings; limit the frequency of intake of salt, simple sugars, and carbonated beverages; increase consumption of fish and white meats at the expense of red meats, processed meats, and cheeses; stay hydrated with water, especially in the morning; engage in moderate aerobic physical activity for about 30 min, even daily (5 days a week). In light of these considerations, the health potential of natural products appears to be associated with the multiple biological activities and pharmacological targets, capable of combating multifactorial diseases such as cardiovascular, neurodegenerative, and some cancers [247].

In the context of oral health, in particular, the antimicrobial, antioxidant, anti-inflammatory, and immunomodulatory potential of nutraceutical substances represent a valid rationale for further studies on oral diseases with infectious, inflammatory, and autoimmune etiology [248].

## 8. Controversial Aspects

The potential of natural compounds in the rehabilitation of oral tissues, such as gum regeneration and tooth repair, is an exciting area of research. However, it is necessary to take into account some important controversies. If, on one hand, preclinical studies, both in vitro and in vivo, along with epidemiological data, allow us to infer a protective role of plant extracts, their actual preventive and therapeutic effects in humans have not yet been unanimously confirmed [188,249]. In particular, clinical data on the chemopreventive potential of flavonoids in oral cancer are still fragmented [250]. The discrepancy between epidemiological data and intervention studies can, at least in part, be explained by the presence of confounding factors that can alter the results of epidemiological studies [123]. For example, smokers may have a lower intake of fruits and vegetables compared to non-smokers; similarly, alcoholics tend to have a lower food intake and, therefore, a lower intake of healthier nutrients [251]. Another limitation concerns the bioavailability of nutraceutical agents in active form in oral tissues/fluids [228]. The mechanisms of action of such compounds in humans may differ from those observed in cell cultures and laboratory animals [106,123]. The lack of pharmacokinetic and pharmacodynamic clinical studies hinders the understanding of this fundamental aspect. To reach effective concentrations at the target site, for example, the oral cavity, the bioactive components of ingested foods must overcome a series of barriers at the gastrointestinal level and resist, in active form, the biotransformation by phase I and II enzymes, first-pass hepatic metabolism, and intestinal microbial flora [252].

Similarly, in terms of therapy, the use of plant extracts for the treatment of oral diseases requires further studies to validate their real clinical efficacy. Randomized, blind clinical studies are lacking to compare them with current standard treatments, using a placebo only if there is no specific treatment available [253].

The information currently available allows for some conclusive considerations. It is also the role of dentistry to promote correct dietary lifestyles: a healthy diet encompasses the synergistic effect of different nutraceutical agents, capable of exerting a protective action against inflammatory and chronic-degenerative pathologies. The efficacy of nutraceutical substances should be verified for each oral disease. The evidence available to date suggests that plant extracts are more effective than individual compounds. As previously illustrated, the various phytochemical substances, by their different pharmacological targets, may be more effective against multifactorial aetiology diseases: additive and/or synergistic effects are likely to maximize the effects of individual compounds on oral health. The possibility of periodic use of mouthwashes based on essential oils as an alternative to chlorhexidine in case of difficulties in proper brushing (psychomotor deficits, post-surgical periods, poor manual dexterity or compliance) seems to be valid. The use of *A. vera* products can be considered a milder alternative to topical cortisone therapy to promote the healing of mild erosive lesions of autoimmune origin (LPO or aphthous stomatitis).

## 9. Conclusions

Natural compounds hold promise in the rehabilitation of oral disease by offering anti-inflammatory, antimicrobial, and regenerative properties, potentially aiding in tissue repair and reducing disease progression. Given such oral health-promoting properties of natural compounds, in dental settings, clinicians assume a relevant role in encouraging patients to adopt healthy lifestyles and dietary habits. Therefore, nutritional recommendations for oral health should be personalized and aligned with valid suggestions for overall health. Specifically, it is particularly effective to encourage patients to increase their consumption of fruits and vegetables and to reduce alcohol intake.

Increasing awareness of the role of nutraceuticals in oral diseases provides the foundation to design novel prophylactic approaches to reduce inflammatory and oxidative phenomena, which may favorably modify their clinical course and progression. However, it must be acknowledged that the positive effects of the nutrients discussed here (and any nutritional recommendations) are largely based on experimental models and only partly corroborated by clinical trials, which are often heterogeneous (using variable amounts of nutrients) and biased due to improper study methodology (e.g., inadequate for sample size), preventing us from reaching conclusions with an acceptable level of evidence.

In the future, it is necessary to evaluate the protective effects of nutraceuticals or isolated nutrients in experimental settings and clinical trials using a methodology adequate to make nutrient comparisons. Until that point has been made, no recommendation can be given for the use of a single nutrient, or a combination, for primary prevention or to complement oral disease treatment in secondary prevention.

At present, we must conclude that, until more reliable data becomes available, there is still the need for cautious optimism, and further studies are required to deepen the mechanisms of action and the real bioavailability of natural compounds.

## Figures and Tables

**Table 4:** Clinical evidence regarding the use of the most effective nutraceutical substances in dentistry.

Oral Diseases	Plant/Insect Species	Substances	References
Dental Caries	*Vitis vinifera*	Grape juice	[130]
	*Theobroma cacao*	Cocoa	[131]
	*Magnolia grandiflora*	Magnolia	[132]
	*Glycyrrhiza glabra*	Licorice	[133]
Periodontal diseases	*Vitis vinifera*	Grape juice	[130]
	*Apis mellifera*	Propolis	[134]
	*Curcuma longa*	Turmeric/Curcumin	[135]
	*Punica sinensis*	Green tea/Catechins	[109]
Oral mucosal lesions	*Perilla frutescens*	Oil	[136]
	*Chamomilla recutita*	Chamomile	[137]
	*Myrtus communis*	Myrtle	[138]
	*Aloe vera*	Aloe	[139]
	*Glycyrrhiza glabra*	Licorice	[140]
	*Acacia erioloba*	Distillate	[141]
Oral candidiasis	*Allium sativum*	Garlic	[142]
	*Ricinus communis*	Castor oil	[143]
	*Melaleuca alternifolia*	Tea tree	[144]
	*Zataria multiflora*	Gel	[145]
	*Punica granatum*	Pomegranate	[146]
Oral lichen planus	*Aloe vera*	Extract/Gel	[147,148]
Oral leukoplakia	*Camellia sinensis*	Black tea/Green tea	[149,150]
	*Rubus* sp.	Gel (Anthocyanins)	[151]
	*Curcuma longa*	Turmeric/Curcumin	[152]
	*Sophora tonkinensis*, *Polygonum bistorta*, *Prunella vulgaris*, *Sonchus brachyotus*, *Dictamnus dasycarpus*, *Dioscorea bulbifera*	Mixture of herbs	[153]
Oral carcinoma	*Camellia sinensis*	Green tea	[154]
	*Rubus* sp.	Gel (Anthocyanins)	[155]
Oral mucositis	*Matricaria recutita*	Chamomile	[156,157]
	*Vaccinium myrtillus*, *Macleaya cordata*, *Echinacea angustifolia*	Mixture of herbs	[158]
	Honey, olive oil, propolis, beeswax	Ointment	[159]
Burning Mouth Syndrome (BMS)	*Capsicum* sp.	Capsaicin	[160,161]
	*Paullinia cupana*, *Trichilia catigua*, *Zingiber officinalis*, *Ptychopetalum olacoides*	Mixture of herbs	[162]
Oral Submucous Fibrosis	*Salvia* sp.	Salvianolic acid	[163]
	*Aloe vera*	Aloe	[164]

**Table 5:** List of the selected granted patents related to oral care products containing natural compounds.

Patent	Jurisdiction *	Title	Publication Date	Reference
US9554986B2	US	Oral care composition	31 January 2017	[234]
US7074391B1	US	Use of olive oil in the preparation of a product for oral hygiene for eliminating or reducing bacterial plaque and/or bacteria in the mouth	11 July 2006	[235]
KR20160041408A	KR	Oral composition containing fermentative extract of galla rhoids as active ingredient	18 April 2016	[236]
KR20180055521A	KR	Composition for prevention or treatment of oral disease comprising icaritin	25 May 2018	[237]
CN113318020A	CN	Multifunctional tooth powder	31 August 2021	[238]
KR20190041801A	KR	Composition for prevention or treatment of oral disease comprising Ginkgolide C	23 April 2019	[239]
KR20180047704A	KR	Composition for prevention or treatment of oral disease comprising Scutellaria baicalensis extract	10 May 2018	[240]
KR20180055519A	KR	Composition for prevention or treatment of oral disease comprising salvianolic acid A	25 May 2018	[241]
KR20180055520A	KR	Composition for prevention or treatment of oral disease comprising neferine	25 May 2018	[242]
JP2006306844A	JP	Oral hygiene composition	9 November 2006	[243]

* US: United States; KR: Republic of Korea; CN: China; JP: Japan.

## Data Availability

No new data were created or analyzed in this study. Data sharing is not applicable to this article.

## References

[1] Jin L.J., Lamster I.B., Greenspan J.S., Pitts N.B., Scully C., Warnakulasuriya S. (2016). Global burden of oral diseases: Emerging concepts, management and interplay with systemic health. Oral Dis..

[2] Welti R., Jones B., Moynihan P., Silva M. (2023). Evidence pertaining to modifiable risk factors for oral diseases: An umbrella review to Inform oral health messages for Australia. Aust. Dent. J..

[3] Genco R.J., Borgnakke W.S. (2013). Risk factors for periodontal disease. Periodontology 2000.

[4] Silva P., Araujo R., Lopes F., Ray S. (2023). Nutrition and Food Literacy: Framing the Challenges to Health Communication. Nutrients.

[5] Clemente-Suarez V.J., Beltran-Velasco A.I., Redondo-Florez L., Martin-Rodriguez A., Tornero-Aguilera J.F. (2023). Global Impacts of Western Diet and Its Effects on Metabolism and Health: A Narrative Review. Nutrients.

[6] Neuhouser M.L. (2019). The importance of healthy dietary patterns in chronic disease prevention. Nutr. Res..

[7] Ma X., Nan F., Liang H., Shu P., Fan X., Song X., Hou Y., Zhang D. (2022). Excessive intake of sugar: An accomplice of inflammation. Front. Immunol..

[8] Billingsley H.E., Carbone S., Lavie C.J. (2018). Dietary Fats and Chronic Noncommunicable Diseases. Nutrients.

[9] Cena H., Calder P.C. (2020). Defining a Healthy Diet: Evidence for The Role of Contemporary Dietary Patterns in Health and Disease. Nutrients.

[10] Garza-Juárez A., Pérez-Carrillo E., Arredondo-Espinoza E.U., Islas J.F., Benítez-Chao D.F., Escamilla-García E. (2023). Nutraceuticals and Their Contribution to Preventing Noncommunicable Diseases. Foods.

[11] Kumar S., Korra T., Thakur R., Arutselvan R., Kashyap A.S., Nehela Y., Chaplygin V., Minkina T., Keswani C. (2023). Role of plant secondary metabolites in defence and transcriptional regulation in response to biotic stress. Plant Stress.

[12] Dutta C., Neeser J.-R., German B.J. (2005). Nutraceutical science and technology. Bioprocesses and Biotechnology for Functional Foods and Nutraceuticals.

[13] Bhattacharjee B., Sandhanam K., Ghose S., Barman D., Sahu R.K. (2024). Market Overview of Herbal Medicines for Lifestyle Diseases. Role of Herbal Medicines: Management of Lifestyle Diseases.

[14] Isola G. (2020). The Impact of Diet, Nutrition and Nutraceuticals on Oral and Periodontal Health. Nutrients.

[15] Isola G. (2020). Current Evidence of Natural Agents in Oral and Periodontal Health. Nutrients.

[16] Winn D.M., Ziegler R.G., Pickle L.W., Gridley G., Blot W.J., Hoover R.N. (1984). Diet in the etiology of oral and pharyngeal cancer among women from the southern United States. Cancer Res..

[17] Chuang S.-C., Jenab M., Heck J.E., Bosetti C., Talamini R., Matsuo K., Castellsague X., Franceschi S., Herrero R., Winn D.M. (2012). Diet and the risk of head and neck cancer: A pooled analysis in the INHANCE consortium. Cancer Causes Control.

[18] Lucenteforte E., Garavello W., Bosetti C., La Vecchia C. (2009). Dietary factors and oral and pharyngeal cancer risk. Oral Oncol..

[19] Ortiz A., Sansinenea E. (2023). Phenylpropanoid Derivatives and Their Role in Plants’ Health and as antimicrobials. Curr. Microbiol..

[20] Dhalaria R., Verma R., Kumar D., Puri S., Tapwal A., Kumar V., Nepovimova E., Kuca K. (2020). Bioactive Compounds of Edible Fruits with Their Anti-Aging Properties: A Comprehensive Review to Prolong Human Life. Antioxidants.

[21] Neelam, Khatkar A., Sharma K.K. (2020). Phenylpropanoids and its derivatives: Biological activities and its role in food, pharmaceutical and cosmetic industries. Crit. Rev. Food Sci. Nutr..

[22] Vogt T. (2010). Phenylpropanoid biosynthesis. Mol. Plant.

[23] Barros J., Dixon R.A. (2020). Plant Phenylalanine/Tyrosine Ammonia-lyases. Trends Plant Sci..

[24] Sabra A., Netticadan T., Wijekoon C. (2021). Grape bioactive molecules, and the potential health benefits in reducing the risk of heart diseases. Food Chem. X.

[25] Di Lorenzo C., Colombo F., Biella S., Stockley C., Restani P. (2021). Polyphenols and Human Health: The Role of Bioavailability. Nutrients.

[26] Kennedy D.O. (2014). Polyphenols and the human brain: Plant “secondary metabolite” ecologic roles and endogenous signaling functions drive benefits. Adv. Nutr..

[27] Pandey K.B., Rizvi S.I. (2009). Plant polyphenols as dietary antioxidants in human health and disease. Oxid. Med. Cell Longev..

[28] Del Bo C., Bernardi S., Marino M., Porrini M., Tucci M., Guglielmetti S., Cherubini A., Carrieri B., Kirkup B., Kroon P. (2019). Systematic Review on Polyphenol Intake and Health Outcomes: Is there Sufficient Evidence to Define a Health-Promoting Polyphenol-Rich Dietary Pattern?. Nutrients.

[29] Phan M.A.T., Paterson J., Bucknall M., Arcot J. (2018). Interactions between phytochemicals from fruits and vegetables: Effects on bioactivities and bioavailability. Crit. Rev. Food Sci. Nutr..

[30] Safe S., Jayaraman A., Chapkin R.S., Howard M., Mohankumar K., Shrestha R. (2021). Flavonoids: Structure-function and mechanisms of action and opportunities for drug development. Toxicol. Res..

[31] Dias M.C., Pinto D., Silva A.M.S. (2021). Plant Flavonoids: Chemical Characteristics and Biological Activity. Molecules.

[32] Flieger J., Flieger W., Baj J., Maciejewski R. (2021). Antioxidants: Classification, Natural Sources, Activity/Capacity Measurements, and Usefulness for the Synthesis of Nanoparticles. Materials.

[33] Patel K.R., Scott E., Brown V.A., Gescher A.J., Steward W.P., Brown K. (2011). Clinical trials of resveratrol. Ann. N. Y. Acad. Sci..

[34] Hasan M., Bae H. (2017). An Overview of Stress-Induced Resveratrol Synthesis in Grapes: Perspectives for Resveratrol-Enriched Grape Products. Molecules.

[35] Iriti M. (2011). Editorial: Introduction to polyphenols, plant chemicals for human health. Mini Rev. Med. Chem..

[36] Guo Y., Li Z., Chen F., Chai Y. (2023). Polyphenols in Oral Health: Homeostasis Maintenance, Disease Prevention, and Therapeutic Applications. Nutrients.

[37] de Moura C.F.G., Noguti J., de Jesus G.P.P., Ribeiro F.A., Garcia F.A., Gollucke A.P., Aguiar O., Ribeiro D.A. (2013). Polyphenols as a chemopreventive agent in oral carcinogenesis: Putative mechanisms of action using in-vitro and in-vivo test systems. Eur. J. Cancer Prev..

[38] De Stefani E., Deneo-Pellegrini H., Boffetta P., Ronco A.L., Aune D., Acosta G., Mendilaharsu M., Brennan P., Ferro G. (2009). Dietary patterns and risk of cancer: A factor analysis in Uruguay. Int. J. Cancer.

[39] Rossi M., Garavello W., Talamini R., Negri E., Bosetti C., Dal Maso L., Lagiou P., Tavani A., Polesel J., Barzan L. (2007). Flavonoids and the risk of oral and pharyngeal cancer: A case-control study from Italy. Cancer Epidemiol. Biomark. Prev..

[40] Vlachojannis C., Magora F., Chrubasik S. (2012). Rise and fall of oral health products with Canadian bloodroot extract. Phytother. Res..

[41] Shankar E., Goel A., Gupta K., Gupta S. (2017). Plant flavone apigenin: An emerging anticancer agent. Curr. Pharmacol. Rep..

[42] Cannataro R., Fazio A., La Torre C., Caroleo M.C., Cione E. (2021). Polyphenols in the Mediterranean Diet: From Dietary Sources to microRNA Modulation. Antioxidants.

[43] Pyrzynska K. (2022). Hesperidin: A Review on Extraction Methods, Stability and Biological Activities. Nutrients.

[44] Kazemi A., Iraji A., Esmaealzadeh N., Salehi M., Hashempur M.H. (2024). Peppermint and menthol: A review on their biochemistry, pharmacological activities, clinical applications, and safety considerations. Crit. Rev. Food Sci. Nutr..

[45] Dabeek W.M., Marra M.V. (2019). Dietary Quercetin and Kaempferol: Bioavailability and Potential Cardiovascular-Related Bioactivity in Humans. Nutrients.

[46] Zoechling A., Reiter E., Eder R., Wendelin S., Liebner F., Jungbauer A. (2009). The flavonoid kaempferol is responsible for the majority of estrogenic activity in red wine. Am. J. Enol. Vitic..

[47] Liu F., Wang Y., Corke H., Zhu H. (2022). Dynamic changes in flavonoids content during congou black tea processing. LWT.

[48] Silva L.C.R.C.e., David J.M., Borges R.d.S.Q., Ferreira S.L.C., David J.P., Reis P.S.d., Bruns R.E. (2014). Determination of Flavanones in Orange Juices Obtained from Different Sources by HPLC/DAD. J. Anal. Methods Chem..

[49] Ribeiro I.A., Ribeiro M.H.L. (2008). Naringin and naringenin determination and control in grapefruit juice by a validated HPLC method. Food Control.

[50] de Oliveira J.R., Camargo S.E.A., de Oliveira L.D. (2019). *Rosmarinus officinalis* L. (rosemary) as therapeutic and prophylactic agent. J. Biomed. Sci..

[51] Munoz-Bernal O.A., Vazquez-Flores A.A., de la Rosa L.A., Rodrigo-Garcia J., Martinez-Ruiz N.R., Alvarez-Parrilla E. (2023). Enriched Red Wine: Phenolic Profile, Sensory Evaluation and In Vitro Bioaccessibility of Phenolic Compounds. Foods.

[52] Rocchetti G., Luisa Callegari M., Senizza A., Giuberti G., Ruzzolini J., Romani A., Urciuoli S., Nediani C., Lucini L. (2022). Oleuropein from olive leaf extracts and extra-virgin olive oil provides distinctive phenolic profiles and modulation of microbiota in the large intestine. Food Chem..

[53] Shabir I., Kumar Pandey V., Shams R., Dar A.H., Dash K.K., Khan S.A., Bashir I., Jeevarathinam G., Rusu A.V., Esatbeyoglu T. (2022). Promising bioactive properties of quercetin for potential food applications and health benefits: A review. Front. Nutr..

[54] Farhan M., Rizvi A. (2023). The Pharmacological Properties of Red Grape Polyphenol Resveratrol: Clinical Trials and Obstacles in Drug Development. Nutrients.

[55] Weiskirchen S., Weiskirchen R. (2016). Resveratrol: How Much Wine Do You Have to Drink to Stay Healthy?. Adv. Nutr..

[56] Bouvier F., Rahier A., Camara B. (2005). Biogenesis, molecular regulation and function of plant isoprenoids. Prog. Lipid Res..

[57] Buhaescu I., Izzedine H. (2007). Mevalonate pathway: A review of clinical and therapeutical implications. Clin. Biochem..

[58] Ashour M., Wink M., Gershenzon J. (2010). Biochemistry of Terpenoids: Monoterpenes, Sesquiterpenes and Diterpenes. Annual Plant Reviews Volume 40: Biochemistry of Plant Secondary Metabolism.

[59] Sadgrove N.J., Padilla-Gonzalez G.F., Phumthum M. (2022). Fundamental Chemistry of Essential Oils and Volatile Organic Compounds, Methods of Analysis and Authentication. Plants.

[60] Katekar V.P., Rao A.B., Sardeshpande V.R. (2023). A hydrodistillation-based essential oils extraction: A quest for the most effective and cleaner technology. Sustain. Chem. Pharm..

[61] Solanki K., Matnani M., Kale M., Joshi K., Bavdekar A., Bhave S., Pandit A. (2005). Transcutaneous absorption of topically massaged oil in neonates. Indian. Pediatr..

[62] Bencze B., Temesfői V., Das S., Papp H., Kaltenecker P., Kuczmog A., Jakab F., Kocsis B., Kőszegi T. (2023). Development of a novel, entirely herbal-based mouthwash effective against common oral bacteria and SARS-CoV-2. BMC Complement. Med. Ther..

[63] Thosar N., Basak S., Bahadure R.N., Rajurkar M. (2013). Antimicrobial efficacy of five essential oils against oral pathogens: An in vitro study. Eur. J. Dent..

[64] Richards D. (2017). Effect of essential oil mouthwashes on plaque and gingivitis. Evid. Based Dent..

[65] Farid Ayad B., Prado R., Dentales D.E., Mateo L.R., Stewart B., BSEng M.G.S., Arvanitidou E., Panagakos P.F.S. (2011). A comparative investigation to evaluate the clinical efficacy of an alcohol-free CPC-containing mouthwash as compared to a control mouthwash in controlling dental plaque and gingivitis: A six-month clinical study on adults in San Jose, Costa Rica. J. Clin. Dent..

[66] Stoeken J.E., Paraskevas S., Van Der Weijden G.A. (2007). The long-term effect of a mouthrinse containing essential oils on dental plaque and gingivitis: A systematic review. J. Periodontol..

[67] Reale S., Biancolillo A., Gasparrini C., Di Martino L., Di Cecco V., Manzi A., Di Santo M., D’Archivio A.A. (2021). Geographical Discrimination of Bell Pepper (*Capsicum annuum*) Spices by (HS)-SPME/GC-MS Aroma Profiling and Chemometrics. Molecules.

[68] Mahleyuddin N.N., Moshawih S., Ming L.C., Zulkifly H.H., Kifli N., Loy M.J., Sarker M.M.R., Al-Worafi Y.M., Goh B.H., Thuraisingam S. (2021). *Coriandrum sativum* L.: A Review on Ethnopharmacology, Phytochemistry, and Cardiovascular Benefits. Molecules.

[69] Keskin I., Gunal Y., Ayla S., Kolbasi B., Sakul A., Kilic U., Gok O., Koroglu K., Ozbek H. (2017). Effects of Foeniculum vulgare essential oil compounds, fenchone and limonene, on experimental wound healing. Biotech. Histochem..

[70] Awada F., Hamade K., Kassir M., Hammoud Z., Mesnard F., Rammal H., Fliniaux O. (2023). Laurus nobilis Leaves and Fruits: A Review of Metabolite Composition and Interest in Human Health. Appl. Sci..

[71] Kennedy D., Okello E., Chazot P., Howes M.J., Ohiomokhare S., Jackson P., Haskell-Ramsay C., Khan J., Forster J., Wightman E. (2018). Volatile Terpenes and Brain Function: Investigation of the Cognitive and Mood Effects of *Mentha* × *Piperita* L. Essential Oil with In Vitro Properties Relevant to Central Nervous System Function. Nutrients.

[72] Feriotto G., Marchetti N., Costa V., Torricelli P., Beninati S., Tagliati F., Mischiati C. (2018). Selected terpenes from leaves of *Ocimum basilicum* L. induce hemoglobin accumulation in human K562 cells. Fitoterapia.

[73] Agliassa C., Maffei M.E. (2018). Origanum vulgare Terpenoids Induce Oxidative Stress and Reduce the Feeding Activity of Spodoptera littoralis. Int. J. Mol. Sci..

[74] Herrera-Calderon O., Saleh A.M., Mahmood A.A.R., Khalaf M.A., Calva J., Loyola-Gonzales E., Tataje-Napuri F.E., Chávez H., Almeida-Galindo J.S., Chavez-Espinoza J.H. (2023). The Essential Oil of *Petroselinum crispum* (Mill) Fuss Seeds from Peru: Phytotoxic Activity and In Silico Evaluation on the Target Enzyme of the Glyphosate Herbicide. Plants.

[75] Pieracci Y., Ciccarelli D., Giovanelli S., Pistelli L., Flamini G., Cervelli C., Mancianti F., Nardoni S., Bertelloni F., Ebani V.V. (2021). Antimicrobial Activity and Composition of Five *Rosmarinus* (*Now Salvia* spp. and Varieties) Essential Oils. Antibiotics.

[76] Fachini-Queiroz F.C., Kummer R., Estevao-Silva C.F., Carvalho M.D., Cunha J.M., Grespan R., Bersani-Amado C.A., Cuman R.K. (2012). Effects of Thymol and Carvacrol, Constituents of *Thymus vulgaris* L. Essential Oil, on the Inflammatory Response. Evid. Based Complement. Altern. Med..

[77] Ferreira M.U. (2022). Alkaloids in Future Drug Discovery. Molecules.

[78] Gutiérrez-Grijalva E.P., López-Martínez L.X., Contreras-Angulo L.A., Elizalde-Romero C.A., Heredia J.B., Swamy M. (2020). Plant Alkaloids: Structures and Bioactive Properties. Plant-Derived Bioactives.

[79] Talapatra S.K., Talapatra B. (2015). Atropine [(±)-Hyoscyamine] and Cocaine (*Ornithine-Derived Alkaloids*). Chemistry of Plant Natural Products: Stereochemistry, Conformation, Synthesis, Biology, and Medicine.

[80] Khan F., Qidwai T., Shukla R.K., Gupta V. (2013). Alkaloids Derived from Tyrosine: Modified Benzyltetrahydroisoquinoline Alkaloids. Natural Products.

[81] Banyal A., Tiwari S., Sharma A., Chanana I., Patel S.K.S., Kulshrestha S., Kumar P. (2023). Vinca alkaloids as a potential cancer therapeutics: Recent update and future challenges. 3 Biotech.

[82] Li F., Jiang T., Li Q., Ling X. (2017). Camptothecin (CPT) and its derivatives are known to target topoisomerase I (Top1) as their mechanism of action: Did we miss something in CPT analogue molecular targets for treating human disease such as cancer?. Am. J. Cancer Res..

[83] Talapatra S.K., Talapatra B. (2015). Ephedrine and Pseudoephedrine (C6–C1 Part Derived from l-Phenylalanine and Nitrogen Derived by Transamination). Chemistry of Plant Natural Products: Stereochemistry, Conformation, Synthesis, Biology, and Medicine.

[84] Chen C., Lin L. (2020). Alkaloids in Diet. Handbook of Dietary Phytochemicals.

[85] Purkiewicz A., Pietrzak-Fiecko R., Sorgel F., Kinzig M. (2022). Caffeine, Paraxanthine, Theophylline, and Theobromine Content in Human Milk. Nutrients.

[86] Mascarenhas A.K., Allen C.M., Loudon J. (2001). The association between Viadent^®^ use and oral leukoplakia. Epidemiology.

[87] della Salute M. (2009). Linee Guida Nazionali per la Promozione Della Salute Orale e la Prevenzione Delle Patologie Orali in Età Adulta.

[88] Gramza-Michałowska A. (2014). Caffeine in tea *Camellia sinensis*—Content, absorption, benefits and risks of consumption. J. Nutr. Health Aging.

[89] Rudolph E., Farbinger A., Konig J. (2012). Determination of the caffeine contents of various food items within the Austrian market and validation of a caffeine assessment tool (CAT). Food Addit. Contam. Part A.

[90] Sharangi A.B. (2009). Medicinal and therapeutic potentialities of tea (*Camellia sinensis* L.)—A review. Food Res. Int..

[91] Dixit T., Pramanic A., Phadtare S., Barde N., Pundir V., Ravindran S. (2023). Identification of Caffeine in Extracts of *Camellia sinensis* by Liquid Chromatography. J. Pharm. Negat. Results.

[92] Wang C., Han J., Pu Y., Wang X. (2022). Tea (*Camellia sinensis*): A Review of Nutritional Composition, Potential Applications, and Omics Research. Appl. Sci..

[93] Zeng W., Zeng Z., Teng J., Rothenberg D.O.N., Zhou M., Lai R., Lai X., Zhao W., Li D., Yan C. (2022). Comparative Analysis of Purine Alkaloids and Main Quality Components of the Three Camellia Species in China. Foods.

[94] dos Santos A.M., Marques L.C., Gonçalves C.P., Marcucci M.C. (2019). Botanical Aspects, Caffeine Content and Antioxidant Activity of *Coffea arabica*. Am. J. Plant Sci..

[95] Bobková A., Demianová A., Poláková K., Capcarová M., Lidiková J., Árvay J., Hegedűsová A., Bobko M., Jurčaga L., Belej Ľ. (2022). Variability of caffeine content in green and roasted Coffea arabica regarding the origin, post-harvest processing, and altitude, and overview of recommended daily allowance. J. Environ. Sci. Health Part B.

[96] Cangeloni L., Bonechi C., Leone G., Consumi M., Andreassi M., Magnani A., Rossi C., Tamasi G. (2022). Characterization of Extracts of Coffee Leaves (*Coffea arabica* L.) by Spectroscopic and Chromatographic/Spectrometric Techniques. Foods.

[97] Meoni G., Luchinat C., Gotti E., Cadena A., Tenori L. (2021). Phenotyping Green and Roasted Beans of Nicaraguan Coffea Arabica Varieties Processed with Different Post-Harvest Practices. Appl. Sci..

[98] Mazzafera P., Crozier A., Magalhães A.C. (1991). Caffeine metabolism in Coffea arabica and other species of coffee. Phytochemistry.

[99] Perrois C., Strickler S.R., Mathieu G., Lepelley M., Bedon L., Michaux S., Husson J., Mueller L., Privat I. (2015). Differential regulation of caffeine metabolism in *Coffea arabica* (Arabica) and *Coffea canephora* (Robusta). Planta.

[100] Júnior P.C.G., dos Santos V.B., Lopes A.S., de Souza J.P.I., Pina J.R.S., Chagas Júnior G.C.A., Marinho P.S.B. (2020). Determination of theobromine and caffeine in fermented and unfermented Amazonian cocoa (*Theobroma cacao* L.) beans using square wave voltammetry after chromatographic separation. Food Control.

[101] Edo G.I., Samuel P.O., Oloni G.O., Ezekiel G.O., Onoharigho F.O., Oghenegueke O., Nwachukwu S.C., Rapheal O.A., Ajokpaoghene M.O., Okolie M.C. (2023). Review on the Biological and Bioactive components of Cocoa (Theobroma Cacao). Insight on Food, Health and Nutrition. Nat. Resour. Hum. Health.

[102] Brunetto M.a.d.R., Gutiérrez L., Delgado Y., Gallignani M., Zambrano A., Gómez Á., Ramos G., Romero C. (2007). Determination of theobromine, theophylline and caffeine in cocoa samples by a high-performance liquid chromatographic method with on-line sample cleanup in a switching-column system. Food Chem..

[103] Ford A., Williams A., de Vries M.S. (2022). New light on the use of Theobroma cacao by Late Classic Maya. Proc. Natl. Acad. Sci. USA.

[104] Picciotti M., Di Vece L., Picciotti V., Lorenzini G. (2013). L’attività antimicrobica dei fitoterapici in odontoiatria. Dent. Cadmos.

[105] Chiu K.C., Shih Y.H., Wang T.H., Lan W.C., Li P.J., Jhuang H.S., Hsia S.M., Shen Y.W., Yuan-Chien Chen M., Shieh T.M. (2021). In vitro antimicrobial and antipro-inflammation potential of honokiol and magnolol against oral pathogens and macrophages. J. Formos. Med. Assoc..

[106] Petti S., Scully C. (2009). Polyphenols, oral health and disease: A review. J. Dent..

[107] Yamanaka A., Kouchi T., Kasai K., Kato T., Ishihara K., Okuda K. (2007). Inhibitory effect of cranberry polyphenol on biofilm formation and cysteine proteases of Porphyromonas gingivalis. J. Periodontal Res..

[108] Ho K.Y., Tsai C.C., Huang J.S., Chen C.P., Lin T.C., Lin C.C. (2001). Antimicrobial activity of tannin components from *Vaccinium vitis-idaea* L.. J. Pharm. Pharmacol..

[109] Hirasawa M., Takada K., Makimura M., Otake S. (2002). Improvement of periodontal status by green tea catechin using a local delivery system: A clinical pilot study. J. Periodontal Res..

[110] Izui S., Sekine S., Maeda K., Kuboniwa M., Takada A., Amano A., Nagata H. (2016). Antibacterial Activity of Curcumin Against Periodontopathic Bacteria. J. Periodontol..

[111] Hu J.P., Takahashi N., Yamada T. (2008). Coptidis Rhizoma inhibits growth and proteases of oral bacteria. Oral Dis..

[112] Ghildiyal R., Prakash V., Chaudhary V., Gupta V., Gabrani R. (2020). Phytochemicals as antiviral agents: Recent updates. Plant-Derived Bioactives: Production, Properties and Therapeutic Applications.

[113] Rathee P., Chaudhary H., Rathee S., Rathee D., Kumar V., Kohli K. (2009). Mechanism of action of flavonoids as anti-inflammatory agents: A review. Inflamm. Allergy Drug Targets.

[114] Kim H.P., Son K.H., Chang H.W., Kang S.S. (2004). Anti-inflammatory plant flavonoids and cellular action mechanisms. J. Pharmacol. Sci..

[115] Willershausen B., Ross A., Forsch M., Willershausen I., Mohaupt P., Callaway A. (2011). The influence of micronutrients on oral and general health. Eur. J. Med. Res..

[116] Basilicata M., Di Lauro M., Campolattano V., Marrone G., Celotto R., Mitterhofer A.P., Bollero P., Di Daniele N., Noce A. (2022). Natural Bioactive Compounds in the Management of Oral Diseases in Nephropathic Patients. Int. J. Environ. Res. Public Health.

[117] Bodet C., Chandad F., Grenier D. (2016). Anti-inflammatory Activity of a High-molecular-weight Cranberry Fraction on Macrophages Stimulated by Lipopolysaccharides from Periodontopathogens. J. Dent. Res..

[118] Bodet C., Chandad F., Grenier D. (2007). Cranberry components inhibit interleukin-6, interleukin-8, and prostaglandin E2 production by lipopolysaccharide-activated gingival fibroblasts. Eur. J. Oral Sci..

[119] Bhattarai G., Poudel S.B., Kook S.H., Lee J.C. (2016). Resveratrol prevents alveolar bone loss in an experimental rat model of periodontitis. Acta Biomater..

[120] Vaziri S., Mojarrab M., Farzaei M.H., Najafi F., Ghobadi A. (2016). Evaluation of anti-aphthous activity of decoction of Nicotiana tabacum leaves as a mouthwash: A placebo-controlled clinical study. J. Tradit. Chin. Med..

[121] Subramanyam R. (2011). Occurrence of recurrent aphthous stomatitis only on lining mucosa and its relationship to smoking—A possible hypothesis. Med. Hypotheses.

[122] Scheid P., Bohadana A., Martinet Y. (2000). Nicotine patches for aphthous ulcers due to Behcet’s syndrome. N. Engl. J. Med..

[123] Iriti M., Varoni E.M. (2013). Chemopreventive potential of flavonoids in oral squamous cell carcinoma in human studies. Nutrients.

[124] Motallebi M., Bhia M., Rajani H.F., Bhia I., Tabarraei H., Mohammadkhani N., Pereira-Silva M., Kasaii M.S., Nouri-Majd S., Mueller A.L. (2022). Naringenin: A potential flavonoid phytochemical for cancer therapy. Life Sci..

[125] Gao W., Wang J., Zhao J. (2022). Describing a modern chemotherapeutic drug prepared by Au nanoparticles to treat the human oral squamous cell carcinoma: A pre-clinical trial study. Inorg. Chem. Commun..

[126] Fath M.K., Nasiri K., Ghasemzadeh S., Nejati S.T., Ghafari N., Masouleh S.S., Dadgar E., Kazemi K.S., Esfahaniani M. (2024). Thymoquinone potentiates anti-cancer effects of cisplatin in oral squamous cell carcinoma via targeting oxidative stress. Chem. Biol. Drug Des..

[127] Sun G.C., Chen H.H., Liang W.-Z., Jan C.-R. (2019). Exploration of the effect of the alkaloid colchicine on Ca^2+^ handling and its related physiology in human oral cancer cells. Arch. Oral Biol..

[128] Zhang H., Xun W., Guo S., Wang X., Liu X. (2022). Anticancer activity of heptazoline against the SCC-15 human oral cancer cells and inhibition of PI3K/AKT signalling pathway. All Life.

[129] Ekor M. (2014). The growing use of herbal medicines: Issues relating to adverse reactions and challenges in monitoring safety. Front. Pharmacol..

[130] Christine D.W. (2009). Grape products and oral health. J. Nutr..

[131] Srikanth R., Shashikiran N., Reddy V.S. (2008). Chocolate mouth rinse: Effect on plaque accumulation and mutans streptococci counts when used by children. J. Indian Soc. Pedod. Prev. Dent..

[132] Campus G., Cagetti M.G., Cocco F., Sale S., Sacco G., Strohmenger L., Lingström P. (2011). Effect of a sugar-free chewing gum containing magnolia bark extract on different variables related to caries and gingivitis: A randomized controlled intervention trial. Caries Res..

[133] Messier C., Epifano F., Genovese S., Grenier D. (2012). Licorice and its potential beneficial effects in common oro-dental diseases. Oral Dis..

[134] Feres M., Figueiredo L.C., Barreto I., Coelho M., Araujo M., Cortelli S.C. (2005). In vitro antimicrobial activity of plant extracts and propolis in saliva samples of healthy and periodontally-involved subjects. J. Int. Acad. Periodontol..

[135] Waghmare P., Chaudhari A., Karhadkar V., Jamkhande A. (2011). Comparative evaluation of turmeric and chlorhexidine gluconate mouthwash in prevention of plaque formation and gingivitis: A clinical and microbiological study. J. Contemp. Dent. Pract..

[136] Hamazaki K., Itomura M., Hamazaki T., Sawazaki S. (2006). Effects of cooking plant oils on recurrent aphthous stomatitis: A randomized, placebo-controlled, double-blind trial. Nutrition.

[137] Ramos-e-Silva M., Ferreira A.F., Bibas R., Carneiro S. (2006). Clinical evaluation of fluid extract of Chamomilla recutita for oral aphthae. J. Drugs Dermatol..

[138] Babaee N., Mansourian A., Momen-Heravi F., Moghadamnia A., Momen-Beitollahi J. (2010). The efficacy of a paste containing Myrtus communis (Myrtle) in the management of recurrent aphthous stomatitis: A randomized controlled trial. Clin. Oral Investig..

[139] Garnick J.J., Singh B., Winkley G. (1998). Effectiveness of a medicament containing silicon dioxide, aloe, and allantoin on aphthous stomatitis. Oral Surg. Oral Med. Oral Pathol. Oral Radiol. Endod..

[140] Moghadamnia A.A., Motallebnejad M., Khanian M. (2009). The efficacy of the bioadhesive patches containing licorice extract in the management of recurrent aphthous stomatitis. Phytother. Res..

[141] Pourahmad M., Rahiminejad M., Fadaei S., Kashafi H. (2010). Effects of camel thorn distillate on recurrent oral aphthous lesions. J. Dtsch. Dermatol. Ges..

[142] Bakhshi M., Taheri J.B., Shabestari S.B., Tanik A., Pahlevan R. (2012). Comparison of therapeutic effect of aqueous extract of garlic and nystatin mouthwash in denture stomatitis. Gerodontology.

[143] Pinelli L.A., Montandon A.A., Corbi S.C., Moraes T.A., Fais L.M. (2013). Ricinus communis treatment of denture stomatitis in institutionalised elderly. J. Oral Rehabil..

[144] Vazquez J.A., Zawawi A.A. (2002). Efficacy of alcohol-based and alcohol-free melaleuca oral solution for the treatment of fluconazole-refractory oropharyngeal candidiasis in patients with AIDS. HIV Clin. Trials.

[145] Amanlou M., Beitollahi J.M., Abdollahzadeh S., Tohidast-Ekrad Z. (2006). Miconazole gel compared with *Zataria multiflora* Boiss. gel in the treatment of denture stomatitis. Phytother. Res..

[146] Vasconcelos L.C., Sampaio M.C., Sampaio F.C., Higino J.S. (2003). Use of Punica granatum as an antifungal agent against candidosis associated with denture stomatitis. Mycoses.

[147] Salazar-Sánchez N., López-Jornet P., Camacho-Alonso F., Sánchez-Siles M. (2010). Efficacy of topical Aloe vera in patients with oral lichen planus: A randomized double-blind study. J. Oral Pathol. Med..

[148] Choonhakarn C., Busaracome P., Sripanidkulchai B., Sarakarn P. (2008). The efficacy of aloe vera gel in the treatment of oral lichen planus: A randomized controlled trial. Br. J. Dermatol..

[149] Li N., Sun Z., Han C., Chen J. (1999). The chemopreventive effects of tea on human oral precancerous mucosa lesions. Proc. Soc. Exp. Biol. Med..

[150] Tsao A.S., Liu D., Martin J., Tang X.-m., Lee J.J., El-Naggar A.K., Wistuba I., Culotta K.S., Mao L., Gillenwater A. (2009). Phase II randomized, placebo-controlled trial of green tea extract in patients with high-risk oral premalignant lesions. Cancer Prev. Res..

[151] Mallery S.R., Zwick J.C., Pei P., Tong M., Larsen P.E., Shumway B.S., Lu B., Fields H.W., Mumper R.J., Stoner G.D. (2008). Topical application of a bioadhesive black raspberry gel modulates gene expression and reduces cyclooxygenase 2 protein in human premalignant oral lesions. Cancer Res..

[152] Hsieh C. (2001). Phase I clinical trial of curcumin, a chemopreventive agent, in patients with high-risk or pre-malignant lesions. Anticancer Res..

[153] Sun Z., Guan X., Li N., Liu X., Chen X. (2010). Chemoprevention of oral cancer in animal models, and effect on leukoplakias in human patients with ZengShengPing, a mixture of medicinal herbs. Oral Oncol..

[154] Schwartz J.L., Baker V., Larios E., Chung F.L. (2005). Molecular and cellular effects of green tea on oral cells of smokers: A pilot study. Mol. Nutr. Food Res..

[155] Shumway B.S., Kresty L.A., Larsen P.E., Zwick J.C., Lu B., Fields H.W., Mumper R.J., Stoner G.D., Mallery S.R. (2008). Effects of a topically applied bioadhesive berry gel on loss of heterozygosity indices in premalignant oral lesions. Clin. Cancer Res..

[156] Mazokopakis E., Vrentzos G., Papadakis J., Babalis D., Ganotakis E. (2005). Wild chamomile (*Matricaria recutita* L.) mouthwashes in methotrexate-induced oral mucositis. Phytomedicine.

[157] Fidler P., Loprinzi C.L., O’Fallon J.R., Leitch J.M., Lee J.K., Hayes D.L., Novotny P., Clemens-Schutjer D., Bartel J., Michalak J.C. (1996). Prospective evaluation of a chamomile mouthwash for prevention of 5-FU-induced oral mucositis. Cancer Interdiscip. Int. J. Am. Cancer Soc..

[158] Pawar D., Neve R., Kalgane S., Riva A., Bombardelli E., Ronchi M., Petrangolini G., Morazzoni P. (2013). SAMITAL^®^ improves chemo/radiotherapy-induced oral mucositis in patients with head and neck cancer: Results of a randomized, placebo-controlled, single-blind Phase II study. Support. Care Cancer.

[159] Abdulrhman M., Samir Elbarbary N., Ahmed Amin D., Saeid Ebrahim R. (2012). Honey and a mixture of honey, beeswax, and olive oil–propolis extract in treatment of chemotherapy-induced oral mucositis: A randomized controlled pilot study. Pediatr. Hematol. Oncol..

[160] de Moraes M., do Amaral Bezerra B.A., da Rocha Neto P.C., de Oliveira Soares A.C.A., Pinto L.P., de Lisboa Lopes Costa A. (2012). Randomized trials for the treatment of burning mouth syndrome: An evidence-based review of the literature. J. Oral Pathol. Med..

[161] Petruzzi M., Lauritano D., De Benedittis M., Baldoni M., Serpico R. (2004). Systemic capsaicin for burning mouth syndrome: Short-term results of a pilot study. J. Oral Pathol. Med..

[162] Spanemberg J.C., Cherubini K., de Figueiredo M.A.Z., Gomes A.P.N., Campos M.M., Salum F.G. (2012). Effect of an herbal compound for treatment of burning mouth syndrome: Randomized, controlled, double-blind clinical trial. Oral Surg. Oral Med. Oral Pathol. Oral Radiol..

[163] Jiang X.-W., Zhang Y., Yang S.-K., Zhang H., Lu K., Sun G.-L. (2013). Efficacy of salvianolic acid B combined with triamcinolone acetonide in the treatment of oral submucous fibrosis. Oral Surg. Oral Med. Oral Pathol. Oral Radiol..

[164] Sudarshan R., Annigeri R.G., Sree Vijayabala G. (2012). Aloe vera in the treatment for oral submucous fibrosis–a preliminary study. J. Oral Pathol. Med..

[165] Marsh P.D. (2010). Microbiology of dental plaque biofilms and their role in oral health and caries. Dent. Clin. N. Am..

[166] Gao Z., Chen X., Wang C., Song J., Xu J., Liu X., Qian Y., Suo H. (2024). New strategies and mechanisms for targeting Streptococcus mutans biofilm formation to prevent dental caries: A review. Microbiol. Res..

[167] Ferrazzano G.F., Amato I., Ingenito A., Zarrelli A., Pinto G., Pollio A. (2011). Plant polyphenols and their anti-cariogenic properties: A review. Molecules.

[168] Ooshima T., Minami T., Aono W., Tamura Y., Hamada S. (1994). Reduction of dental plaque deposition in humans by oolong tea extract. Caries Res..

[169] Jones C., Woods K., Whittle G., Worthington H., Taylor G. (1999). Sugar, drinks, deprivation and dental caries in 14-year-old children in the north west of England in 1995. Community Dent. Health.

[170] Wynn W., Haldi J., Law M.L. (1960). Influence of the ash of the cacao bean on the cariogenicity of a high-sucrose diet. J. Dent. Res..

[171] Verakaki E., Duggal M. (2003). A comparison of different kinds of European chocolates on human plaque pH. Eur. J. Paediatr. Dent..

[172] Goultschin J., Palmon S., Shapira L., Brayer L., Gedalia I. (1991). Effect of glycyrrhizin-containing toothpaste on dental plaque reduction and gingival health in humans: A pilot study. J. Clin. Periodontol..

[173] Steinberg D., Sgan-Cohen H., Stabholz A., Pizanty S., Segal R., Sela M. (1989). The anticariogenic activity of glycyrrhizin: Preliminary clinical trials. Isr. J. Dent. Sci..

[174] Hu C.h., He J., Eckert R., Wu X.y., Li L.n., Tian Y., Lux R., Shuffer J.A., Gelman F., Mentes J. (2011). Development and evaluation of a safe and effective sugar-free herbal lollipop that kills cavity-causing bacteria. Int. J. Oral Sci..

[175] Peters M., Tallman J., Braun T., Jacobson J. (2010). Clinical reduction of S. mutans in pre-school children using a novel liquorice root extract lollipop: A pilot study. Eur. Arch. Paediatr. Dent..

[176] Varma R., Ross C.N. (2017). Liquorice: A root cause of secondary hypertension. JRSM Open.

[177] Könönen E., Kumar P.S. (2015). Bacteriology of Periodontal Diseases. Molecular Medical Microbiology.

[178] Mysak J., Podzimek S., Sommerova P., Lyuya-Mi Y., Bartova J., Janatova T., Prochazkova J., Duskova J. (2014). Porphyromonas gingivalis: Major periodontopathic pathogen overview. J. Immunol. Res..

[179] Antezack A., Etchecopar-Etchart D., La Scola B., Monnet-Corti V. (2023). New putative periodontopathogens and periodontal health-associated species: A systematic review and meta-analysis. J. Periodontal Res..

[180] Adline V.D., Ashwath B., Anitha V., Shanmugam M. (2021). Aggregatibacter actinomycetemcomitans—A periodontopathogen. IP Int. J. Periodontol. Implantol..

[181] Zhang J., Yu J., Dou J., Hu P., Guo Q. (2021). The Impact of Smoking on Subgingival Plaque and the Development of Periodontitis: A Literature Review. Front. Oral Health.

[182] Kushiyama M., Shimazaki Y., Murakami M., Yamashita Y. (2009). Relationship between intake of green tea and periodontal disease. J. Periodontol..

[183] Oliveira S.M.A.d., Torres T.C., Pereira S.L.d.S., Mota O.M.d.L., Carlos M.X. (2008). Effect of a dentifrice containing Aloe vera on plaque and gingivitis control: A double-blind clinical study in humans. J. Appl. Oral Sci..

[184] Hebbal M., Ankola A.V., Sharma R., Johri S. (2012). Effectiveness of herbal and fluoridated toothpaste on plaque and gingival scores among residents of a working women’s hostel—A randomised controlled trial. Oral Health Prev. Dent..

[185] Shetty S., Bose A., Sridharan S., Satyanarayana A., Rahul A. (2013). A clinico-biochemical evaluation of the role of a herbal (Ayurvedic) immunomodulator in chronic periodontal disease: A pilot study. Oral Health Dent. Manag..

[186] Samuels N., Grbic J.T., Saffer A.J., Wexler I.D., Williams R.C. (2012). Effect of an herbal mouth rinse in preventing periodontal inflammation in an experimental gingivitis model: A pilot study. Compend. Contin. Educ. Dent..

[187] Grbic J., Wexler I., Celenti R., Altman J., Saffer A. (2011). A phase II trial of a transmucosal herbal patch for the treatment of gingivitis. J. Am. Dent. Assoc..

[188] Varoni E.M., Lodi G., Sardella A., Carrassi A., Iriti M. (2012). Plant polyphenols and oral health: Old phytochemicals for new fields. Curr. Med. Chem..

[189] Rivera C., Muñoz-Pastén M., Núñez-Muñoz E., Hernández-Olivos R. (2022). Recurrent Aphthous Stomatitis Affects Quality of Life. A Case-Control Study. Clin. Cosmet. Investig. Dent..

[190] Mortazavi H., Safi Y., Baharvand M., Rahmani S. (2016). Diagnostic Features of Common Oral Ulcerative Lesions: An Updated Decision Tree. Int. J. Dent..

[191] Akintoye S.O., Greenberg M.S. (2014). Recurrent aphthous stomatitis. Dent. Clin. N. Am..

[192] Tarakji B., Gazal G., Al-Maweri S.A., Azzeghaiby S.N., Alaizari N. (2015). Guideline for the diagnosis and treatment of recurrent aphthous stomatitis for dental practitioners. J. Int. Oral Health.

[193] Babaee N., Zabihi E., Mohseni S., Moghadamnia A.A. (2012). Evaluation of the therapeutic effects of Aloe vera gel on minor recurrent aphthous stomatitis. Dent. Res. J..

[194] Das S.K., Das V., Gulati A.K., Singh V.P. (1989). Deglycyrrhizinated liquorice in aphthous ulcers. J. Assoc. Physicians India.

[195] Martin M.D., Sherman J., van der Ven P., Burgess J. (2008). A controlled trial of a dissolving oral patch concerning glycyrrhiza (licorice) herbal extract for the treatment of aphthous ulcers. Gen. Dent..

[196] Vila T., Sultan A.S., Montelongo-Jauregui D., Jabra-Rizk M.A. (2020). Oral Candidiasis: A Disease of Opportunity. J. Fungi.

[197] Dadar M., Tiwari R., Karthik K., Chakraborty S., Shahali Y., Dhama K. (2018). Candida albicans—Biology, molecular characterization, pathogenicity, and advances in diagnosis and control—An update. Microb. Pathog..

[198] Sanchez-Martinez C., Perez-Martin J. (2001). Dimorphism in fungal pathogens: Candida albicans and Ustilago maydis--similar inputs, different outputs. Curr. Opin. Microbiol..

[199] Lu S.Y. (2021). Oral Candidosis: Pathophysiology and Best Practice for Diagnosis, Classification, and Successful Management. J. Fungi.

[200] Tiwari A.V., Dangore-Khasbage S. (2024). Oral Thrush: An Entity With a Diagnostic Dilemma. Cureus.

[201] Rai A., Misra S.R., Panda S., Sokolowski G., Mishra L., Das R., Lapinska B. (2022). Nystatin Effectiveness in Oral Candidiasis Treatment: A Systematic Review & Meta-Analysis of Clinical Trials. Life.

[202] Patel K.K., Sehgal V.S., Kashfi K. (2022). Molecular targets of statins and their potential side effects: Not all the glitter is gold. Eur. J. Pharmacol..

[203] Čēma I., Kakar J., Dzudzilo M., Murovska M. (2023). Immunological Aspects of EBV and Oral Mucosa Interactions in Oral Lichen Planus. Appl. Sci..

[204] Lodi G., Varoni E., Salis A., Franchini R. (2011). Oral lichen planus and lichenoid lesions. A guide for the dentist. Dent. Cadmos.

[205] Leong X.Y., Gopinath D., Syeed S.M., Veettil S.K., Shetty N.Y., Menon R.K. (2023). Comparative Efficacy and Safety of Interventions for the Treatment of Oral Lichen Planus: A Systematic Review and Network Meta-Analysis. J. Clin. Med..

[206] Thongprasom K., Carrozzo M., Furness S., Lodi G. (2011). Interventions for treating oral lichen planus. Cochrane Database Syst. Rev..

[207] Chainani-Wu N., Silverman S., Reingold A., Bostrom A., Mc Culloch C., Lozada-Nur F., Weintraub J. (2007). A randomized, placebo-controlled, double-blind clinical trial of curcuminoids in oral lichen planus. Phytomedicine.

[208] Laskaris G. (2023). Potentially Malignant Disorders. Periodontal Manifestations of Local and Systemic Diseases: Color Atlas and Text.

[209] Kumari P., Debta P., Dixit A. (2022). Oral Potentially Malignant Disorders: Etiology, Pathogenesis, and Transformation Into Oral Cancer. Front. Pharmacol..

[210] Rubert A., Bagan L., Bagan J.V. (2021). Retraction: Oral leukoplakia, a clinical-histopathological study in 412 patients. J. Clin. Exp. Dent..

[211] Halder A., Raychowdhury R., Ghosh A., De M. (2005). Black tea (*Camellia sinensis*) as a chemopreventive agent in oral precancerous lesions. J. Environ. Pathol. Toxicol. Oncol..

[212] Badwelan M., Muaddi H., Ahmed A., Lee K.T., Tran S.D. (2023). Oral Squamous Cell Carcinoma and Concomitant Primary Tumors, What Do We Know? A Review of the Literature. Curr. Oncol..

[213] Surh Y.-J. (2003). Cancer chemoprevention with dietary phytochemicals. Nat. Rev. Cancer.

[214] Yang Y., Ye W.L., Zhang R.N., He X.S., Wang J.R., Liu Y.X., Wang Y., Yang X.M., Zhang Y.J., Gan W.J. (2021). The Role of TGF-beta Signaling Pathways in Cancer and Its Potential as a Therapeutic Target. Evid. Based Complement. Altern. Med..

[215] Galeone C., Tavani A., Pelucchi C., Turati F., Winn D.M., Levi F., Yu G.-P., Morgenstern H., Kelsey K., Dal Maso L. (2010). Coffee and tea intake and risk of head and neck cancer: Pooled analysis in the international head and neck cancer epidemiology consortium. Cancer Epidemiol. Biomark. Prev..

[216] Wallace T.C., Bailey R.L., Blumberg J.B., Burton-Freeman B., Chen C.y.O., Crowe-White K.M., Drewnowski A., Hooshmand S., Johnson E., Lewis R. (2019). Fruits, vegetables, and health: A comprehensive narrative, umbrella review of the science and recommendations for enhanced public policy to improve intake. Crit. Rev. Food Sci. Nutr..

[217] Turati F., Galeone C., La Vecchia C., Garavello W., Tavani A. (2011). Coffee and cancers of the upper digestive and respiratory tracts: Meta-analyses of observational studies. Ann. Oncol..

[218] Ide R., Fujino Y., Hoshiyama Y., Mizoue T., Kubo T., Pham T.-M., Shirane K., Tokui N., Sakata K., Tamakoshi A. (2007). A prospective study of green tea consumption and oral cancer incidence in Japan. Ann. Epidemiol..

[219] Li X., Yu C., Guo Y., Bian Z., Shen Z., Yang L., Chen Y., Wei Y., Zhang H., Qiu Z. (2019). Association between tea consumption and risk of cancer: A prospective cohort study of 0.5 million Chinese adults. Eur. J. Epidemiol..

[220] Truong V.L., Jeong W.S. (2021). Cellular Defensive Mechanisms of Tea Polyphenols: Structure-Activity Relationship. Int. J. Mol. Sci..

[221] Hildebrand J.S., Patel A.V., McCullough M.L., Gaudet M.M., Chen A.Y., Hayes R.B., Gapstur S.M. (2013). Coffee, tea, and fatal oral/pharyngeal cancer in a large prospective US cohort. Am. J. Epidemiol..

[222] Radoï L., Paget-Bailly S., Menvielle G., Cyr D., Schmaus A., Carton M., Guida F., Cénée S., Sanchez M., Guizard A.-V. (2013). Tea and coffee consumption and risk of oral cavity cancer: Results of a large population-based case-control study, the ICARE study. Cancer Epidemiol..

[223] Filippini T., Malavolti M., Borrelli F., Izzo A.A., Fairweather-Tait S.J., Horneber M., Vinceti M. (2020). Green tea (*Camellia sinensis*) for the prevention of cancer. Cochrane Database Syst. Rev..

[224] Liao Y.H., Chou W.Y., Chang C.W., Lin M.C., Wang C.P., Lou P.J., Chen T.C. (2023). Chemoprevention of oral cancer: A review and future perspectives. Head Neck.

[225] Grigolato R., Bizzoca M.E., Calabrese L., Leuci S., Mignogna M.D., Lo Muzio L. (2020). Leukoplakia and Immunology: New Chemoprevention Landscapes?. Int. J. Mol. Sci..

[226] Yoon A.J., Shen J., Santella R.M., Philipone E.M., Wu H.-C., Eisig S.B., Blitzer A., Close L.G., Zegarelli D.J. (2012). Topical application of green tea polyphenol (−) epigallocatechin-3-gallate for prevention of recurrent oral neoplastic lesions. J. Orofac. Sci..

[227] Ugalde C.M., Liu Z., Ren C., Chan K.K., Rodrigo K.A., Ling Y., Larsen P.E., Chacon G.E., Stoner G.D., Mumper R.J. (2009). Distribution of anthocyanins delivered from a bioadhesive black raspberry gel following topical intraoral application in normal healthy volunteers. Pharm. Res..

[228] Pulito C., Cristaudo A., Porta C.L., Zapperi S., Blandino G., Morrone A., Strano S. (2020). Oral mucositis: The hidden side of cancer therapy. J. Exp. Clin. Cancer Res..

[229] Jicman Stan D., Sarbu M.I., Fotea S., Nechifor A., Balan G., Anghele M., Vasile C.I., Niculet E., Sarbu N., Rebegea L.F. (2022). Oral Mucositis Induced by Chemoradiotherapy in Head and Neck Cancer-A Short Review about the Therapeutic Management and the Benefits of Bee Honey. Medicina.

[230] Lionel D., Christophe L., Marc A., Jean-Luc C. (2006). Oral mucositis induced by anticancer treatments: Physiopathology and treatments. Ther. Clin. Risk Manag..

[231] Worthington H.V., Clarkson J.E., Bryan G., Furness S., Glenny A.-M., Littlewood A., McCabe M.G., Meyer S., Khalid T., Riley P. (2011). Interventions for preventing oral mucositis for patients with cancer receiving treatment. Cochrane Database Syst. Rev..

[232] Aravindhan R., Vidyalakshmi S., Kumar M.S., Satheesh C., Balasubramanium A.M., Prasad V.S. (2014). Burning mouth syndrome: A review on its diagnostic and therapeutic approach. J. Pharm. Bioallied Sci..

[233] Chhabra A.K., Sune R., Reche A. (2023). Oral Submucous Fibrosis: A Review of the Current Concepts in Management. Cureus.

[234] Harvey N. (2017). Oral Care Composition. U.S. Patent.

[235] Hernandez M.A. (2006). Use of Olive Oil in the Preparation of a Product for Oral Hygiene for Eliminating or Reducing Bacterial Plaque and/or Bacteria in the Mouth. U.S. Patent.

[236] Young L.J., Chul K.H., Mong L.C., Tai L.G., Kook L.K. (2016). Oral Composition Containing Fermentative Extract of Galla Rhoids as Active Ingredient.

[237] Heon Y.J. (2018). Composition for Prevention or Treatment of Oral Disease Comprising Icaritin.

[238] Liang X. (2021). Multifunctional Tooth Powder.

[239] Yu J., Sanghwa L. (2019). Composition for Prevention or Treatment of Oral Disease Comprising Ginkgolide C.

[240] Jung Y., Hwa L.S. (2018). Composition for Prevention or Treatment of Oral Disease Comprising Scutellaria Baicalensis Extract.

[241] Heon Y.J., Hwa L.S. (2018). Composition for Prevention or Treatment of Oral Disease Comprising Salvianolic Acid A.

[242] Heon Y.J., Hwa L.S. (2018). Composition for Prevention or Treatment of Oral Disease Comprising Neferine.

[243] Maruyama T., Kitagori H. (2006). Oral Hygiene Composition.

[244] Sette S., Le Donne C., Piccinelli R., Arcella D., Turrini A., Leclercq C., Group I.-S.S. (2011). The third Italian national food consumption survey, INRAN-SCAI 2005-06—Part 1: Nutrient intakes in Italy. Nutr. Metab. Cardiovasc. Dis..

[245] Who J., Consultation F.E. (2003). Diet, nutrition and the prevention of chronic diseases. World Health Organ. Tech. Rep. Ser..

[246] Riccardi G., Giosuè A., Calabrese I., Vaccaro O. (2022). Dietary recommendations for prevention of atherosclerosis. Cardiovasc. Res..

[247] Mohd Sairazi N.S., Sirajudeen K.N.S. (2020). Natural Products and Their Bioactive Compounds: Neuroprotective Potentials against Neurodegenerative Diseases. Evid. Based Complement. Altern. Med..

[248] Malcangi G., Patano A., Ciocia A.M., Netti A., Viapiano F., Palumbo I., Trilli I., Guglielmo M., Inchingolo A.D., Dipalma G. (2023). Benefits of Natural Antioxidants on Oral Health. Antioxidants.

[249] Moynihan P.J. (2002). Dietary advice in dental practice. Br. Dent. J..

[250] Forni C., Rossi M., Borromeo I., Feriotto G., Platamone G., Tabolacci C., Mischiati C., Beninati S. (2021). Flavonoids: A Myth or a Reality for Cancer Therapy?. Molecules.

[251] McClure J.B., Divine G., Alexander G., Tolsma D., Rolnick S.J., Stopponi M., Richards J., Johnson C.C. (2009). A comparison of smokers’ and nonsmokers’ fruit and vegetable intake and relevant psychosocial factors. Behav. Med..

[252] Rein M.J., Renouf M., Cruz-Hernandez C., Actis-Goretta L., Thakkar S.K., da Silva Pinto M. (2013). Bioavailability of bioactive food compounds: A challenging journey to bioefficacy. Br. J. Clin. Pharmacol..

[253] Tzimas K., Antoniadou M., Varzakas T., Voidarou C. (2024). Plant-Derived Compounds: A Promising Tool for Dental Caries Prevention. Curr. Issues Mol. Biol..

